# Detection of novel Plasmodium falciparum haplotypes under treatment pressure in paediatric severe malaria

**DOI:** 10.1099/mgen.0.001386

**Published:** 2025-05-09

**Authors:** Balotin Fogang, Emilie Guillochon, Claire Kamaliddin, Gino Agbota, Sem Ezinmegnon, Maroufou Jules Alao, Philippe Deloron, Gwladys Bertin, Antoine Claessens

**Affiliations:** 1 LPHI, CNRS, INSERM, University of Montpellier, Montpellier, France; 2 Université Paris Cité, MERIT, IRD, Paris, France; 3 Institut de Recherche Clinique du Bénin (IRCB), Abomey-Calavi, Benin; 4 Paediatric Department, Mother and Child University and Hospital Center (CHU-MEL), Cotonou, Benin

**Keywords:** genetic complexity, parasite clearance, *Plasmodium falciparum*, severe malaria

## Abstract

**Background.** In Africa, the clearance time for *Plasmodium falciparum* severe malaria varies significantly, likely due to the complexity of *P. falciparum* infections and the sequestration phenomenon exhibited by this parasite. This study aims to evaluate different methods to study the intra-host dynamics of polyclonal infections during parasite clearance under antimalarial treatment. Additionally, it seeks to determine the association between parasite clearance rate following artesunate or quinine treatment and the genetic complexity of *P. falciparum* in Beninese children with severe malaria.

**Methods.** Sixty-five *P. falciparum* severe malaria individuals diagnosed by microscopy and treated with artesunate or quinine were sampled every 8 h for 24 h. Using whole-genome sequencing (WGS) data, we estimated the multiplicity of infection (MOI) with three algorithms (*Fws*, THE REAL McCOIL and RoH). We then characterized the *P. falciparum* genetic complexity in WGS-identified polyclonal infections using amplicon sequencing (AmpSeq) on DNA extracted from plasma and the red blood cell pellet.

**Results.** AmpSeq demonstrated greater sensitivity in detecting multiple genomes within isolates compared to WGS methods. The MOI from AmpSeq was significantly higher in red blood cell pellets compared to plasma (2.4 vs. 1.8 distinct microhaplotypes per isolate). However, at parasitaemia over 1,000 parasites per microlitre, the same MOI was detected in both plasma and pellet samples in 85.4% of the isolates. We observed a high variability in parasite clearance rate among participants, but it was not associated with parasite MOI at diagnosis. Interestingly, in 60.9% of participants, previously undetected microhaplotypes appeared in circulation 16 h after treatment initiation.

**Conclusion.** These findings demonstrate that combining different haplotyping techniques effectively determines parasite genetic complexity. Additionally, plasma can be effectively used for parasite genotyping at sufficient parasitaemia levels. The parasite clearance rate of severe malaria is independent of parasite MOI. However, genotyping a single blood sample upon hospital admission does not capture the full spectrum of parasite genotypes present in the infection.

**Impact Statement**

This study provides new insights into the genetic complexity and clearance dynamics of Plasmodium falciparum in children with severe malaria under antimalarial treatment. By comparing whole-genome sequencing and amplicon sequencing methods, we show that AmpSeq is more sensitive for detecting minority parasite genotypes. Our findings reveal that parasite clearance is not associated with multiplicity of infection at admission and that genotyping a single timepoint underestimates within-host diversity due to sequestration. This highlights the limitations of current genotyping practices and underscores the importance of time-series sampling to fully capture the complexity of infections, particularly in regions where drug resistance is not yet established.

## Data Summary

All whole-genome sequencing data generated and analysed in this study have been deposited in the European Nucleotide Archive under the study accession number PRJEB2136, with individual accession numbers in Table S1. The raw reads from the amplicon sequencing (AmpSeq) data are publicly available on Zenodo (https://zenodo.org/records/13224728), with corresponding individual accession numbers provided in Table S3. The raw data and complete AmpSeq results are included as Tables S1 and 2, respectively.

## Introduction

*Plasmodium falciparum* is the predominant malaria species and a major contributor to child mortality in Africa. Many children infected with *P. falciparum* succumb to the disease before even reaching a hospital or clinic. Among those who are admitted with severe malaria and receive parenteral antimalarial treatment, ~5% do not survive, highlighting the urgent need for effective interventions [1]. Since 2011, the World Health Organization (WHO) has recommended intravenous artesunate as the first-line antimalarial treatment for severe malaria [2], citing its potential to reduce severe malaria mortality rates by 22.5% compared to quinine [3]. Nevertheless, in cases where artesunate is unavailable, alternative severe malaria treatment such as intramuscular artemether or intravenous quinine is recommended [2].

Despite the widespread recommendation of artemisinin-based combination therapies (ACTs) as the frontline treatment for malaria, reports of *P. falciparum* resistance to ACTs have risen in Asia [4,5] and more recently in Africa [6,7]. Indeed, artemisinin resistance is characterized by the slow clearance of parasites *in vivo*, which is a result of reduced drug susceptibility in ring-stage parasites driven by mutations in the *P. falciparum Kelch13* gene [8]. Since the introduction of ACT in 2005, no PfKelch13 mutation associated with artemisinin resistance has been identified in Benin [9,10].

Although the PfKelch13 polymorphisms are the strongest predictor of *in vivo* parasite clearance time under treatment with artesunate, this time can be affected by other factors such as the age of the host, host-acquired immunity, initial parasitaemia levels and the developmental stages of the parasite [11,13]. Although controversial, studies have shown a link between the genetic complexity of *P. falciparum* and delayed parasite clearance in Africa. Indeed, children infected with multiple strains had nearly a threefold increase in treatment failure compared to their age mates infected with a single strain [14]. However, low multiplicity of infection (MOI) at baseline was associated with detectable parasitaemia at 72 h post-ACT treatment [15]. The association between antimalarial parasite clearance rate and parasite genetic complexity in individuals with severe malaria (SM) remains to be explored.

In SM, the delay in parasite clearance could also be attributed to the significant sequestration of mature *P. falciparum* parasitized erythrocytes within the tissue capillaries. Mathematical modelling using plasma histidine-rich protein 2 (PfHRP2) levels and parasitaemia have indicated that the number of sequestered parasites is substantially higher than what is detected in the peripheral blood [16,18]. Indeed, in falciparum malaria, parasitized red blood cells circulate in the peripheral blood for only one-third of the 48-h asexual cycle [19]; for the remainder of the cycle, they are sequestered in the venules and capillaries. Because the circulating parasitaemia is highly dependent on the parasite developmental stage, the amount of *Plasmodium* DNA in plasma is more accurate than parasitaemia for diagnostic differentiating severe from uncomplicated malaria [20]. Other studies have also demonstrated that plasma can be used to detect and quantify the *Plasmodium* DNA by Polymerase Chain Reaction (PCR) [21,22]. However, whether genomic DNA (gDNA) extracted from plasma could be used as material to reliably estimate the MOI is not known. Although the plasma may contain less parasite DNA than within red blood cells (RBCs), it is unaffected by sequestration and could theoretically contain parasite DNA released from prior schizont ruptures of all the parasite genotypes present within an individual. However, there is currently no evidence regarding the sensitivity of *Plasmodium* genotyping when using plasma samples as the DNA source.

Various sequencing approaches have recently been used to assess the complexity of *P. falciparum*, quantified as the number of parasite genotypes within an isolate [23]. These methods include whole-genome sequencing (WGS)-based techniques using diverse metrics such as fraction of within-sample (*Fws*) [24], THE REAL McCOIL [25] and runs of homozygosity (RoH) [26], which elucidate intra-host parasite diversity by analysing genome-wide variations across multiple loci. *Fws* is a metric characterizing within-host diversity and its relationship to population-level diversity, with theoretical values ranging from 0 to 1. The RoH indicates genetic relatedness between two or more genotypes within an isolate by identifying long blocks of haplotypes that have been inherited from the same parent. THE REAL McCOIL is a statistical approach that uses data from all infections to simultaneously estimate allelic frequency and the number of distinct genotypes in an isolate. Amplicon sequencing (AmpSeq), a technique targeting highly polymorphic loci, has emerged as a novel tool capable of detecting minority microhaplotypes with within-sample frequencies as low as 0.1% [27]. Indeed, the strength of sequencing targeted non-repetitive regions that harbour extensive SNPs lies in the fact that all SNPs within an amplicon are linked by a single sequence read, enabling direct microhaplotype identification [27]. Therefore, this study aims to evaluate the association between parasite clearance rate and multiplicity of infection in Beninese children undergoing treatment for severe malaria, using a variety of sequencing methods and biological materials.

## Methods

### Study design

Ethical clearance was obtained from ‘Comité d’Ethique de la Recherche CER_ISBA Benin’ (clearance n°90, 06 June 2016, and clearance n°38, 16 May 2014). As recommended by the Technical Expert Group on Malaria Chemotherapy in 2008 [28], the sample size for the surveillance of antimalarial efficacy should be determined using classical statistical methods based on an expected treatment failure in the study population, a level of confidence of 95% and a precision of 5%. As the treatment failure rate of severe malaria was unknown in this study population, a clinical failure of 50% was assumed. For the study to be representative, a minimum sample of 50 patients was required, regardless of the rate of failure as recommended.

During the high malaria transmission season in Southern Benin, children under the age of 6 diagnosed with severe malaria infection at the ‘Centre Hospitalier et Universitaire de la Mère et de l’Enfant-Lagune (CHU-MEL) de Cotonou’ were enrolled in the study in both 2014 and 2016, as described in [29,29]. Prior to their participation, written informed consent from parents or guardians was obtained. Malaria infection was diagnosed in febrile patients by detecting *P. falciparum* on a Giemsa-stained thick blood smear. Parasite density was estimated by counting the number of parasites per at least 200 white blood cells (WBCs) and assuming a standard total blood WBC count of 8,000 WBC per microlitre. To ensure consistency and accuracy, microscopy was performed by two experienced microscopists. All discordant results were read by a third microscopist, whose results were taken as final. Diagnosis and classification of severe malaria were carried out by WHO guidelines for the management of severe malaria [2]. In brief, cerebral malaria (CM) was characterized by impaired consciousness, indicated by a Blantyre score <3, after excluding other causes of coma. Severe malaria anaemia (SMA) was identified by a haemoglobin level <5 g dL^−1^ or a haematocrit level <15%. Severe non-cerebral malaria (SNCM) was defined by the features of severe malaria, excluding CM involvement. Furthermore, clinical appreciation was at the discretion of the admitting physician.

Approximately 5 ml of blood was collected from all participants using EDTA-coated tubes before the administration of quinine (in 2014) or artesunate (in 2016) therapy (H0). Subsequently, three additional blood samples were taken at +8 h (H8), +16 h (H16) and +24 h (H24) post-treatment initiation to capture parasite dynamics.

### DNA extraction

Plasma was separated from total blood cells by centrifugation at 1,500 r.p.m. for 5 min and immediately stored at −20 °C. RBC pellet was obtained by depleting WBCs using a gradient-based separation technique Ficoll according to the manufacturer’s instructions (GE Healthcare Life Science). *P. falciparum* gDNA was extracted from both 200 µl of RBC pellets and 200 µl of plasma using the DNEasy Blood kit (QIAGEN) according to the manufacturer’s instructions.

### Complexity of infection using WGS data

WGS was performed by the Malaria Genomic Epidemiology Network (MalariaGEN) and the samples are part of the *P. falciparum* Community Project accessible at https://www.malariagen.net/project/p-falciparum-community-project/. The raw reads are available on the European Nucleotide Archive server under the accession numbers specified in Table S1 (available in the online Supplementary Material). Variant Calling Files (VCF) were generated by MalariaGEN as previously described [30]. Briefly, reads were mapped to *P. falciparum* 3D7 v3 reference genome using bwa mem, and the resulting BAM files were subjected to cleaning using Picard tools and GATK. SNPs and indels were called using GATK HaplotypeCaller, with only the core genome considered for analysis.

For downstream analysis, bcftools v1.13 [31] and bedtools v2.30 [32] were used for file manipulation. Samples with at least 50% coverage of the genome at 5× were considered. *Fws* metrics were computed using the moimix R package, as previously described (https://github.com/bahlolab/moimix). An *Fws* value <0.95 was indicative of a polyclonal infection. THE REAL McCOIL categorical method was used to estimate the complexity of the infection (MOI) as described by Chang *et al*. [25] (https://github.com/Greenhouse-Lab/THEREALMcCOIL). Long RoH and the analysis of heterozygosity in mixed samples were carried out using a custom Python script based on Pearson *et al*. [26].

### DNA preparation and AmpSeq

Three AmpSeq markers, including the genes for conserved plasmodium membrane protein (*cpmp*, PF3D7_0104100), conserved plasmodium protein (*cpp*, PF3D7_1475800) and apical membrane antigen (*ama1-D3*, PF3D7_1133400), were amplified using nested PCR, as described in [33]. Briefly, primary PCRs were conducted in multiplex for *cpmp/ama1-D3* and monoplex for *cpp*. Nested PCRs were subsequently performed individually for each marker. All amplifications were carried out using the KAPA HiFi HotStart Ready Mix (Roche) on an Eppendorf Mastercycler Nexus Thermocycler. The quality and quantity of nested PCR products were assessed through gel electrophoresis. Following this, nested-PCR products from each sample were combined in the following proportions: 11 µl of cpmp, 8 µl of *cpp* and 4 µl of *ama1-D3*, for a total of 25 µl. PCR primers and amplification conditions are shown in Table S2.

Library preparations and amplicon sequencing were performed by the Genseq platform of the Labex CeMEB (Montpellier). Merged PCR products were purified, and sequencing adapters and multiplexing indices were linked by PCR. Indexed products were purified and pooled, and quality was verified by electrophoresis using the Fragment Analyzer system (Agilent Biotechnologies). PCR products resulting from RBC pellet or plasma gDNA were separated in two different plates to prevent the impact of the difference in DNA concentration on the sequencing quality. Finally, amplicon libraries were sequenced on an Illumina MiSeq system in paired-end mode (2×300 cycles, 300 bp). The raw AmpSeq reads are available on Zenedo (https://zenodo.org/records/13224728), with corresponding individual accession numbers provided in Table S3.

Markers haplotypes were reconstructed using the HaplotypR R package [27], accessible at https://github.com/lerch-a/HaplotypR. Briefly, the quality of raw reads data was assessed using FastQC. Reads corresponding to each marker were demultiplexed based on primer sequences, with subsequent truncation of primers. Reads were trimmed following sequence quality and then fused. SNPs and microhaplotypes were called with default parameters, requiring a minimum coverage of 3 reads per microhaplotype, at least 25 reads coverage per sample and a within-host microhaplotype frequency ≥1%. Microhaplotype sequences that differed by less than two SNPs were deemed identical. The relative frequency of each microhaplotype in an isolate was calculated by dividing the number of reads for each microhaplotype by the total number of reads for that isolate. For downstream analysis, the marker showing the highest number of microhaplotypes in an individual was retained. The same marker was chosen for the four time points in an individual. The raw data for AmpSeq are presented in Table S3.

### Statistical analysis

All statistical analyses were performed using R software version 4.3.2 [34]. Median comparisons of two quantitative variables were conducted using the Mann–Whitney test, while mean comparisons were assessed using the unpaired t-test. The correlation between two quantitative variables was assessed using the Spearman correlation test. For categorical variables, proportions were analysed using the chi-squared test. We used linear interpolation to estimate the time required for a 50% reduction in parasitaemia, referred to as the extrapolated parasite clearance half-life (PC_50%_). The clearance curve for each microhaplotype (parasite genotype detected by AmpSeq) was calculated by multiplying the parasitaemia at each time point by the relative frequency of each microhaplotype. Shannon entropy was used to characterize the complexity of microhaplotypes within an isolate obtained from AmpSeq.

## Results

### Characteristics of the study population

**Fig. 1.**
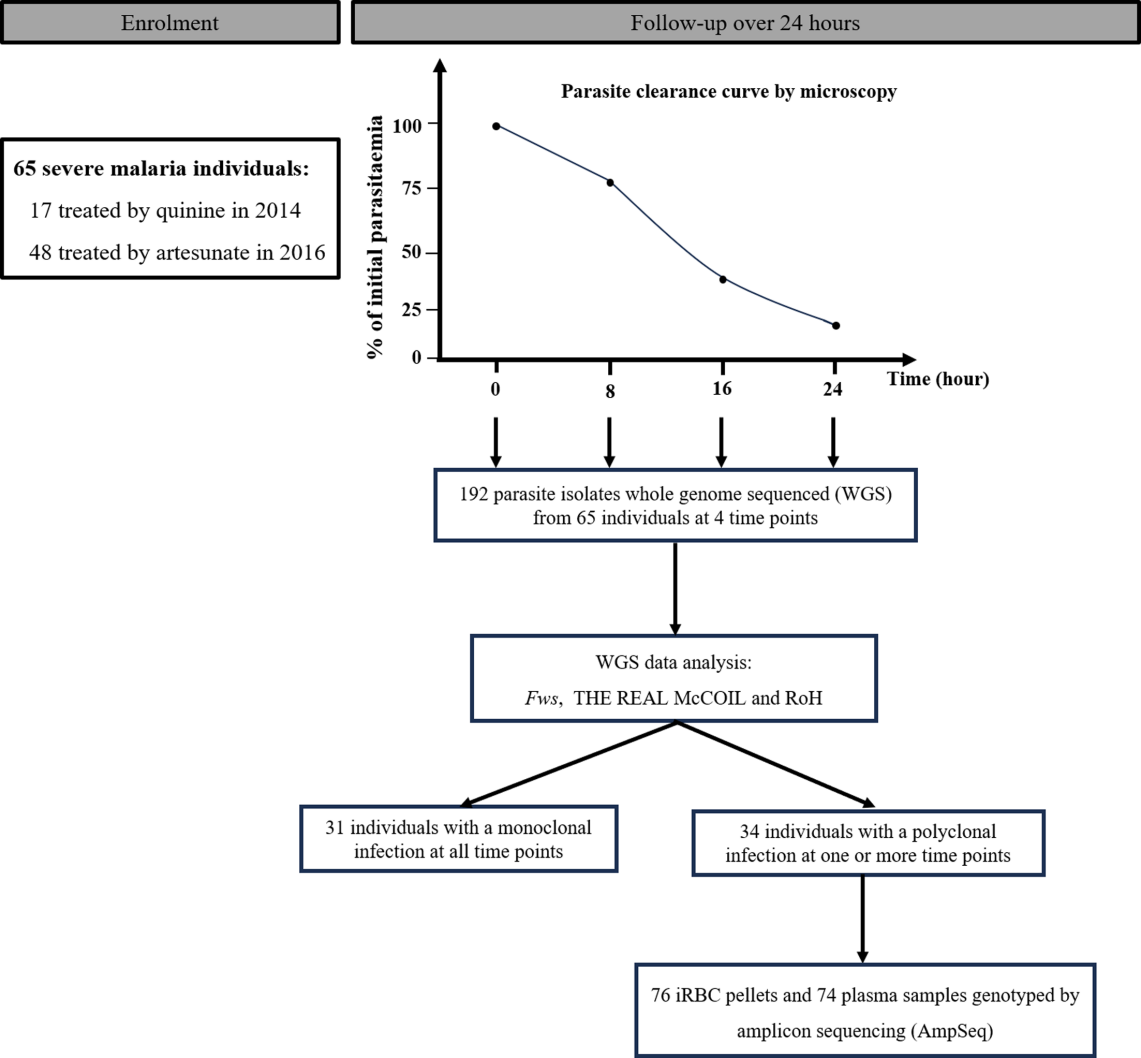
Study flow diagram. Sixty-five severe malaria patients were sampled at the start of treatment and every 8 h for 24 h afterwards. While 65 individuals were enrolled, not all participants had samples collected at all 4 time points. This was due to the clinical challenge of managing children with severe malaria, such as early mortality, medical instability or insufficient blood volume at certain time points. As a result, the total number of analysed samples was 230 instead of the expected 260. Out of 230 isolates submitted for WGS, 192 yielded high-quality genomes. The MOI was determined from parasite whole-genome sequences. From 34 individuals with at least 1 polyclonal time point, AmpSeq was performed on plasma and RBC pellet samples. A total of 76 RBC pellet isolates and 74 plasma gDNA isolates from 23 individuals were successfully genotyped.

During the years 2014 and 2016, a total of 65 children aged 0 to 5 years old suffering from severe malaria and admitted at CHU-MEL in Benin were recruited. Of these participants, 17 received quinine in 2014, while 48 received artesunate in 2016 (Fig. 1). These individuals were classified as 26 CM, 16 SNCM and 23 SMA (Table S1). The median age and sex ratios of participants were comparable between quinine- and artemisinin-treated groups. However, the geometric mean parasitaemia in 2014 was significantly higher than in 2016 (*P*<0.0001). The demographic and parasitological characteristics of the participants at enrolment are provided in Table 1. Blood samples were also collected on arrival at the hospital and every 8 h for 24 h (Fig. 1).

**Table 1.** Demographic and clinical background of the study participants

Characteristics	Artesunate	Quinine	***P*-value**
Number of participants (*N*=65)	48	17	
Sex ratio (M/F)	1.9 (27/14)NA=7	1.3 (9/7)NA=1	0.5506
Median age, months (range)	30 (5–60)NA=5	36 (9–59)	0.2144
Geometric mean parasitaemia per microlitre (range)	6.66×10⁴ (4.05×10² – 2.15×10⁶)	1.32×10⁶ (1.54×10⁴ – 3.20×10⁶)	<0.0001

### Determining the MOI based on WGS

We first aimed to characterize the MOI using WGS. A total of 230 RBC pellet samples at each time point were whole-genome sequenced, resulting in 192 good-quality genomes (Fig. 1 and Table S1). To assess the MOI from these genomes, three algorithms were tested: *F_WS_*, THE REAL McCOIL and RoH. All three algorithms showed robust correlation (Appendix 1). From here onwards, any genome with *F_WS_* >0.95 was considered monoclonal.

### Determining the MOI based on AmpSeq using gDNA from RBC pellets and plasma

We used the amplicon sequencing approach (AmpSeq) to assess MOI from paired RBC pellets and plasma samples obtained from the same individuals. The rationale was that the RBC pellet represents circulating parasites at a certain time point, while plasma could contain parasite DNA from previous schizont ruptures that is independent of sequestration. As described in the ‘Methods’ section, three markers (*cpmp*, *cpp* and *ama1*) were PCR-amplified and sequenced, yielding an average of 43,400 reads per sample per loci (Tables S3 and S4). We observed a high correlation between the MOI values across the three markers (*r*>0.60, *P*<0.0001) (Fig. S1). The marker showing the highest number of microhaplotypes in an individual was retained (*cpmp*, *cpp* and *ama1* in 34.8%, 34.8% and 30.4% of individuals, respectively, Table S3). AmpSeq was successfully performed at each time point sample from 23 individuals with a polyclonal infection, as determined by WGS methods. Sixty-nine isolates were successfully genotyped in both RBC pellets and plasma gDNA, seven were genotyped in RBC pellets gDNA only and five in plasma gDNA only (Table S1).

**Fig. 2.**
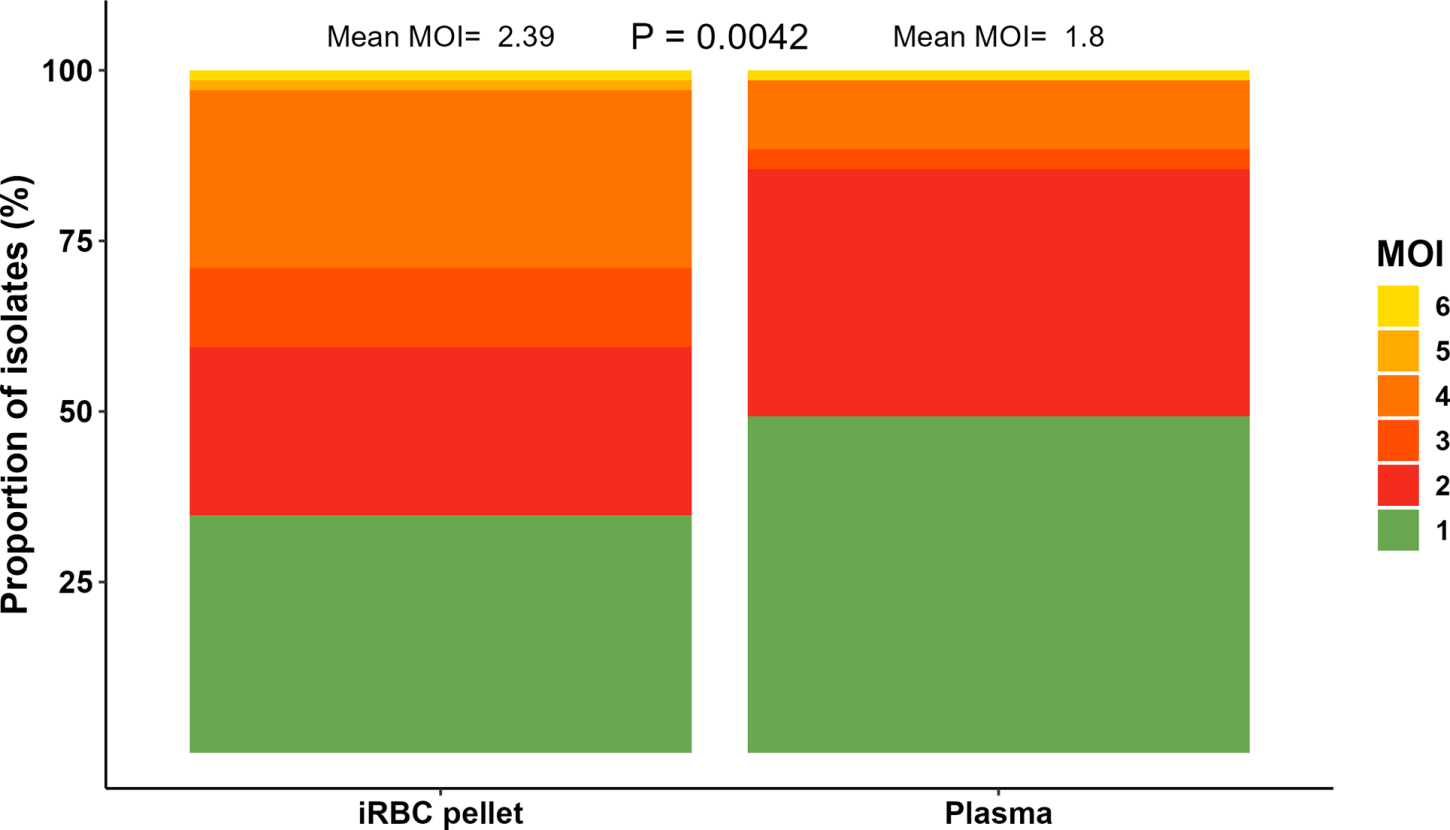
Comparison of the multiplicity of the infection determined using AmpSeq with gDNA from paired RBC pellet and plasma samples. The MOI per isolate, indicated as a colour scale, is the number of distinct microhaplotypes detected per isolate.

In terms of sequence diversity, a total of 65 unique microhaplotypes were detected across all isolates. Among these, 98.5% (64) were identified in pellets, while 70.8% (46) were found in plasma. Furthermore, 19 microhaplotypes (29.2%) were exclusively present in pellets, while only 1 microhaplotype was found in plasma. The mean MOI per isolate was significantly higher in RBC pellets compared to plasma (2.4 and 1.8 for pellets and plasma, respectively, *P*=0.0042) (Fig. 2). Among isolates from the same individual, 66.7% (46/69) exhibited an identical number of genotypes detected when using gDNA from both RBC pellets and plasma. Furthermore, at parasitaemia over 1,000 parasites per microlitre, the same MOI was detected in both plasma and pellet samples in 85.4% of the isolates (Fig. S2). This indicates the influence of parasite load on the sensitivity of AmpSeq when using plasma as the source of DNA. However, these data also show that plasma can be effectively used for parasite genotyping at relatively high parasitaemia levels.

**Fig. 3.**
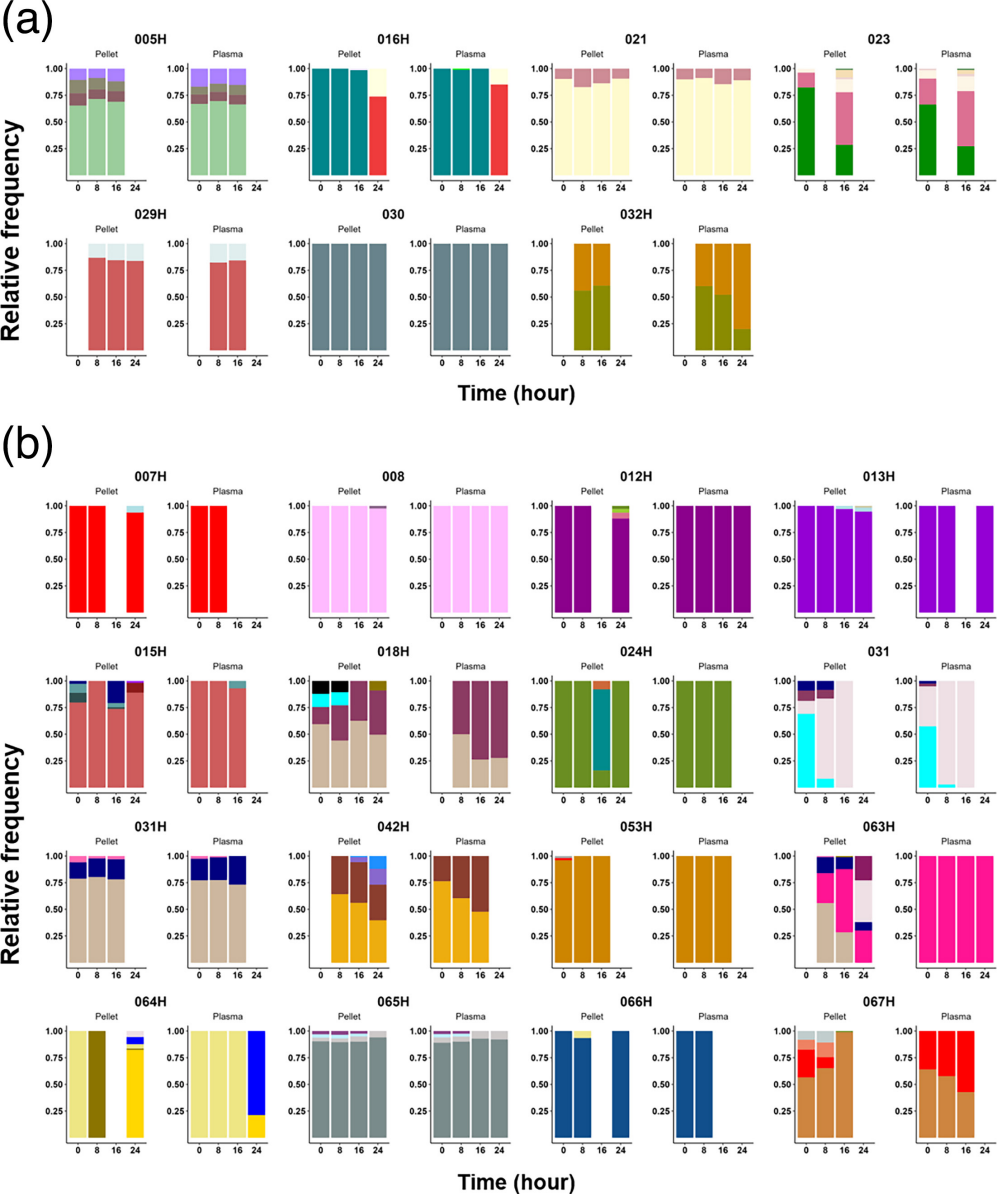
Relative proportion of microhaplotypes within patients, from AmpSeq using paired gDNA extracted from RBC pellets and plasma samples. (a) Patients with the same microhaplotype profile between plasma and pellet gDNA. (b) Patients with more microhaplotypes (higher MOI) were detected within RBC pellet gDNA compared to plasma. Each colour indicates a unique microhaplotype within the whole population.

By visualizing the evolution of clonality at each time point (0 h, 8 h, 16 h and 24 h) during the 24 h after treatment initiation (Fig. 3), 7 infections showed the same MOI profile from RBC pellet and plasma over time (Fig. 3a), while in 16 infections, more microhaplotypes were detected in RBC pellet than in plasma (Fig. 3b). Taken together, AmpSeq using gDNA from RBC pellets is more sensitive compared to AmpSeq using gDNA from plasma at low-range parasitaemia. From here onwards, the AmpSeq MOI was defined as the number of microhaplotypes detected with gDNA from RBC pellets.

### Comparison of AmpSeq and WGS-based methods for determining MOI from pellet gDNA

**Fig. 4.**
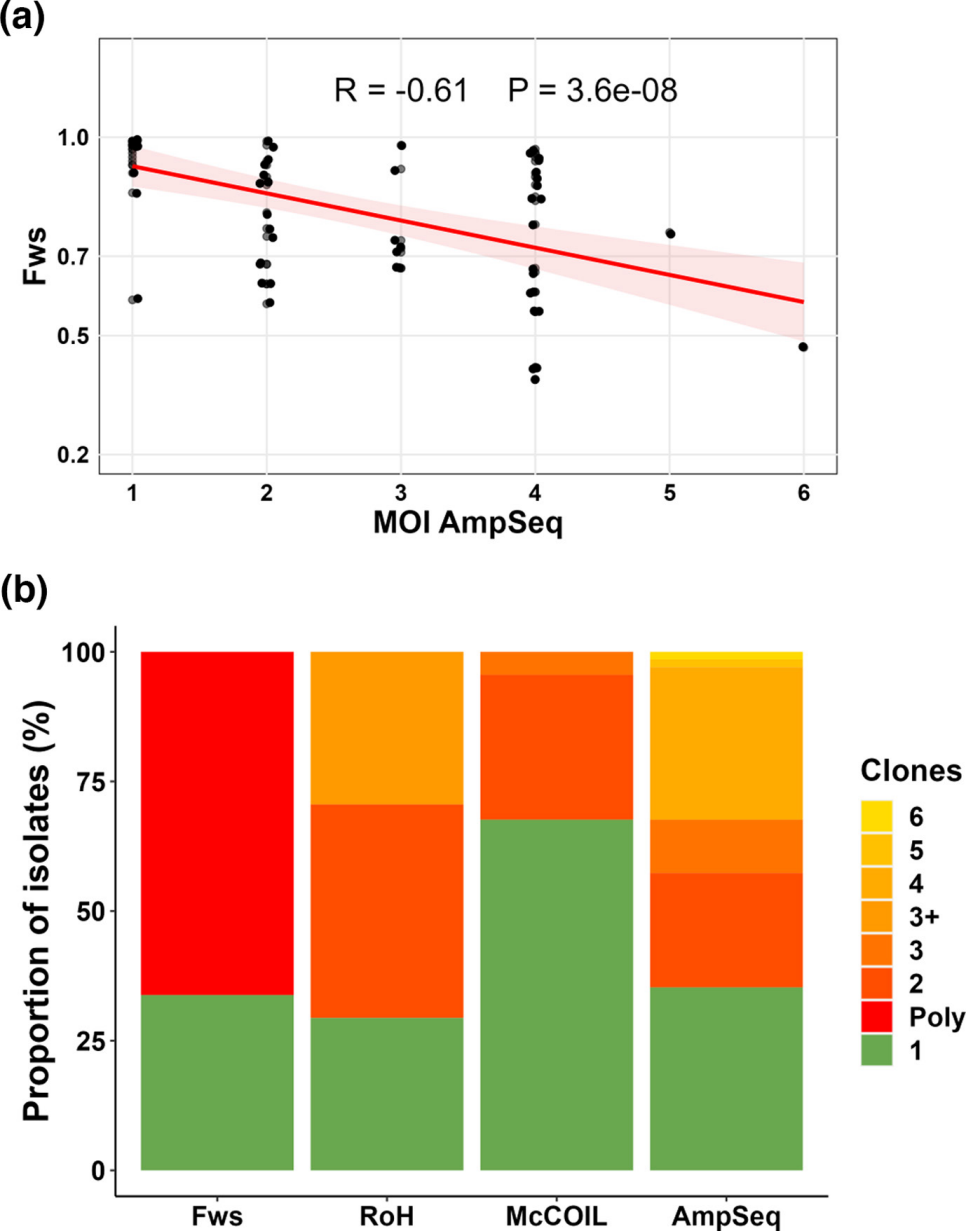
Comparison of AmpSeq to different WGS-based algorithms for determining the MOI. (a) Correlation between *Fws* and AmpSeq. (b) Proportion of isolates according to clonality in different genotyping techniques. Only individuals with at least one time point at *Fws* <0.95 were included in this analysis.

A total of 68 isolates had MOI data available for both AmpSeq and WGS. We found a good correlation between AmpSeq and the different WGS-based methods (*r*=−0.61, *r*=0.47 and *r*=−0.59 for *Fws*, THE REAL McCOIL and RoH, respectively) (Fig. 4). In addition, 80.8% and 79.3% of the isolates showed concordant results (either monoclonal or polyclonal) between AmpSeq and *Fws* or AmpSeq and RoH, respectively (Fig. 4b). However, the AmpSeq method was able to detect up to six genotypes in an isolate, indicating greater sensitivity of AmpSeq in detecting multiple genomes in an isolate compared to WGS-based methods (Fig. 4b). Furthermore, AmpSeq was the only method capable of quantifying the proportion of each genotype identified within an isolate. Together, these findings showed that the combination of different haplotyping techniques can be used to characterize genetic complexity quantitatively and qualitatively within an isolate. The MOI of a polyclonal isolate was then defined from the AmpSeq data.

### Variability in parasite clearance rate among individuals and high rate of rebound in parasitaemia during antimalarial treatment

**Fig. 5.**
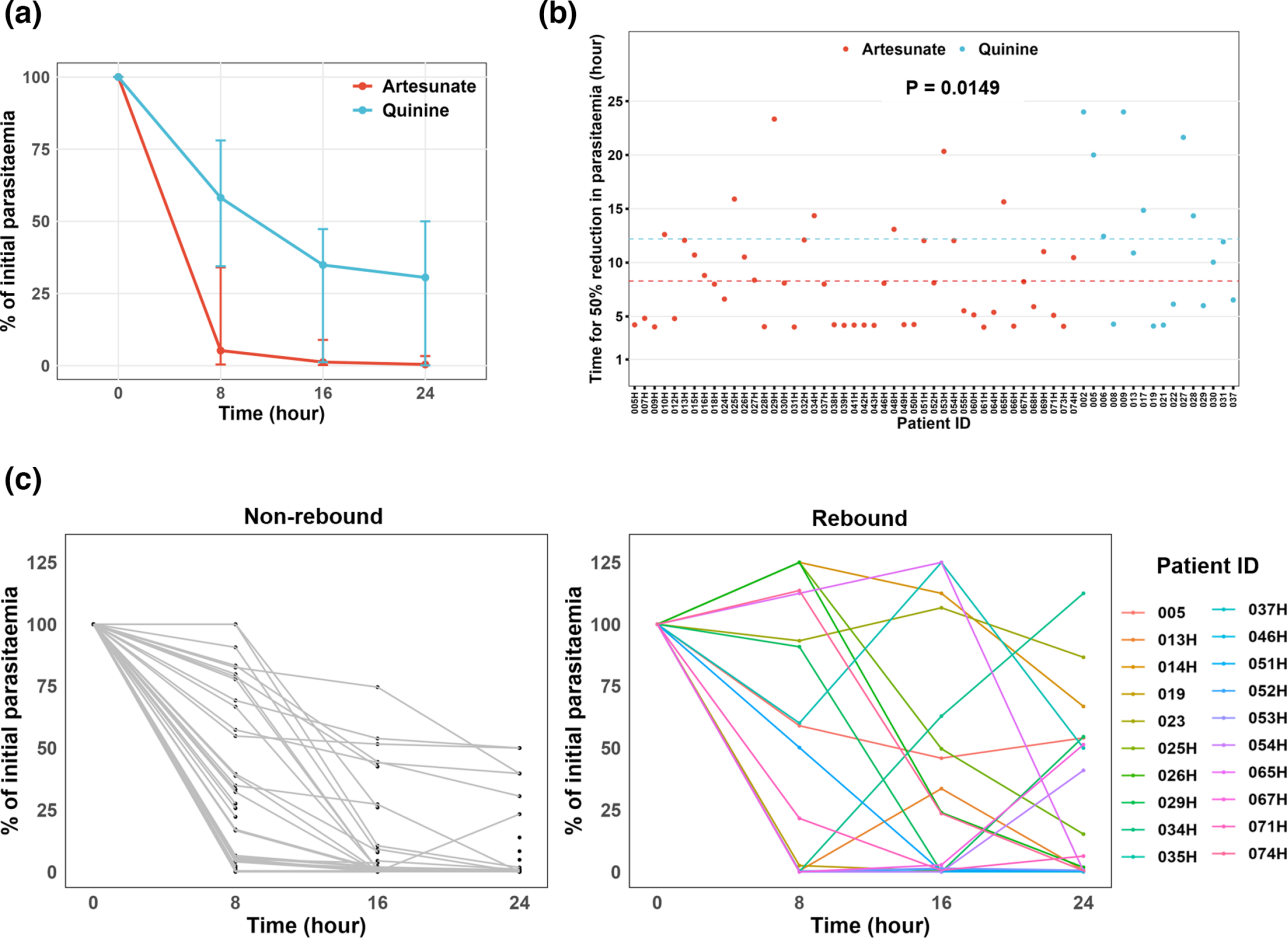
Parasite clearance curves of severe malaria cases treated with artesunate or quinine. (a) Parasite clearance curve according to antimalarial treatment. (b) Time for 50% reduction in parasitaemia according to treatment. (c) Parasite clearance curve highlighting the rebound in parasitaemia post-antimalarial treatment.

Having established the most sensitive method for MOI determination, we then aimed to characterize the clearance rate within the first 24 h of treatment. Median parasite clearance time was faster in children treated with artesunate compared to those treated with quinine (Fig. 5a), with the mean PC_50%_ of 8.3 and 12.2 h, respectively (Fig. 5b). There was a high variability in parasite clearance half-life within both treatment groups, ranging from 4.1 to 23.3 h for artesunate and 4.0 to 24.0 h for quinine (Fig. 5b). Of the 48 individuals treated with artesunate, 95.6% (43/45) had a PC_50%_ below 16 h, compared to 75% (12/16) of individuals treated with quinine (Fig. 5b). The PC_50%_ could not be determined for four individuals (023, 014 h, 029 h and 063 h) due to a non-significant decrease in parasitaemia (<50% reduction) between the first and last time points (Data S2). Parasite clearance curves are within the same range as previous studies using quinine or artesunate [35] (Data S2). These curves indicated substantial variation in initial clearance rates between individuals, highlighting the complex dynamics of parasite response to treatment in the early stage of infection management.

We took advantage of the time-series to investigate intra-host competition in polyclonal infections. Interestingly, we observed a ‘rebound’ in the parasitaemia determined by microscopy reading. We defined rebound as an increase in parasite densities by at least 100 parasites per microlitre between two time points and observed this phenomenon in 34.8% (20/65) of individuals (Fig. 5c). Specifically, within the artesunate-treated group, 35.4% (17/48) of individuals experienced a rebound, while in the quinine-treated group, the rebound rate was 17.6% (3/17). These data indicate the potential release of sequestered parasites during antimalarial treatment.

### *P. falciparum* clearance rate is not related to MOI at the onset of the treatment

**Fig. 6.**
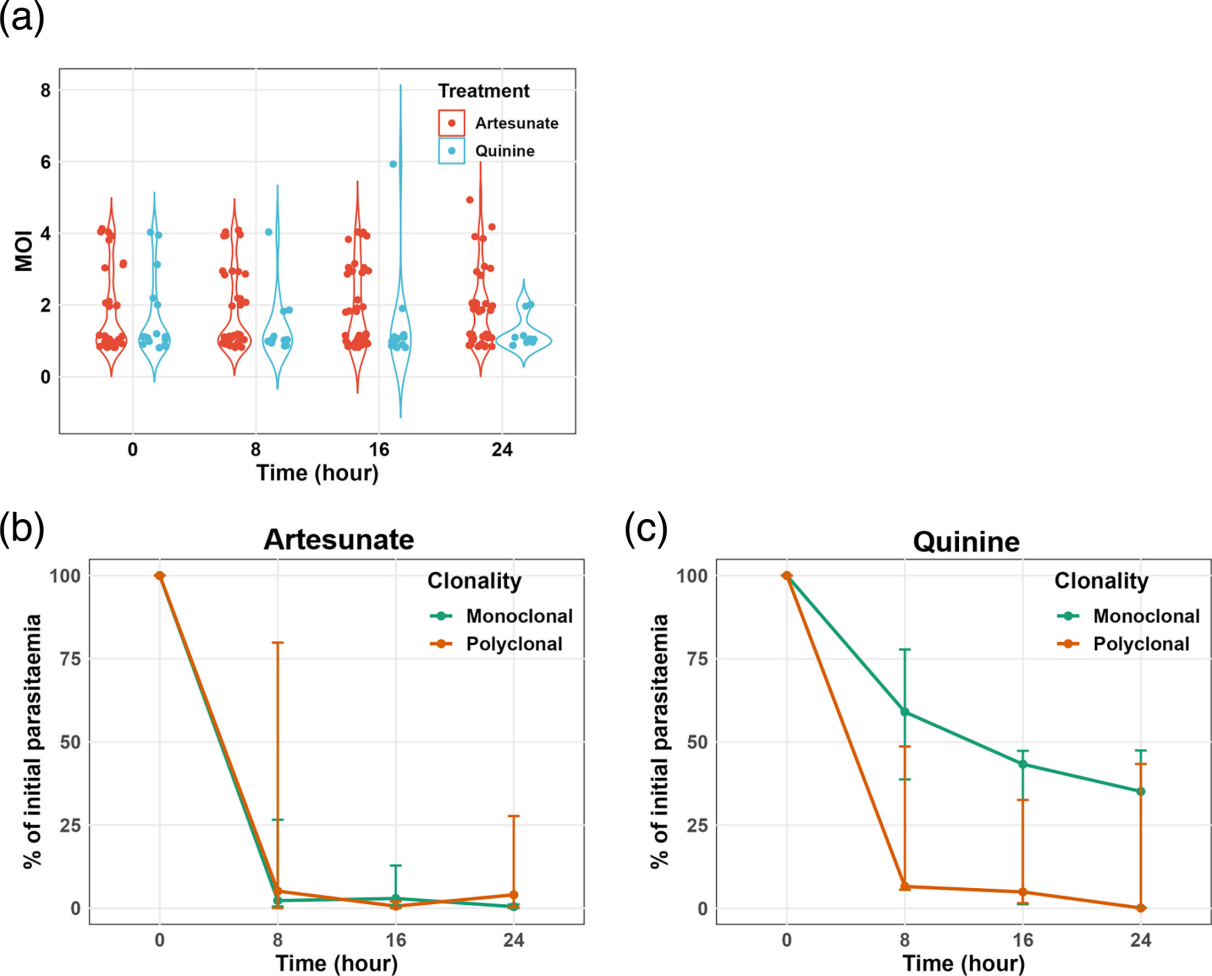
Dynamic of parasite clearance to parasite multiplicity of the infection. (a) Dynamic of MOI determined by AmpSeq throughout the antimalarial treatment. Parasite clearance rate according to clonality at enrolment (H0) for artesunate (b) and quinine (c) treatment groups.

To determine whether the variability in the clearance rate of *P. falciparum* depends on the number of parasite genotypes in the infection, the dynamics of parasite clearance were compared between individuals with polyclonal and monoclonal infections during the first 24 h of severe malaria management under treatment pressure. Independently of the treatment administered, the mean MOI was relatively stable throughout the 24 h period (Fig. 6a). In individuals treated with artesunate, *P. falciparum* clearance showed no significant difference between polyclonal and monoclonal infections (Fig. 6b). In contrast, although non-significant, treatment with quinine resulted in faster *P. falciparum* clearance in polyclonal infections (mean PC_50%_ of 4.3) compared to monoclonal infections (mean PC_50%_ of 12.4) (*P*=0.1393) (Fig. 6c). Part of this difference may be attributed to the higher median parasitaemia at enrolment in individuals treated with quinine and having monoclonal infections compared to those with polyclonal infections (2.44×10⁶ vs 1.52×10⁶ parasites per microlitre, *P*=0.1924). In addition, there was no correlation between MOI at enrolment and parasite clearance rate (*r*=−0.06, *P*=0.6494). These data show that the * P. falciparum* clearance rate does not depend on the initial parasite MOI.

### Sporadic detection of novel microhaplotypes during antimalarial treatment

**Fig. 7.**
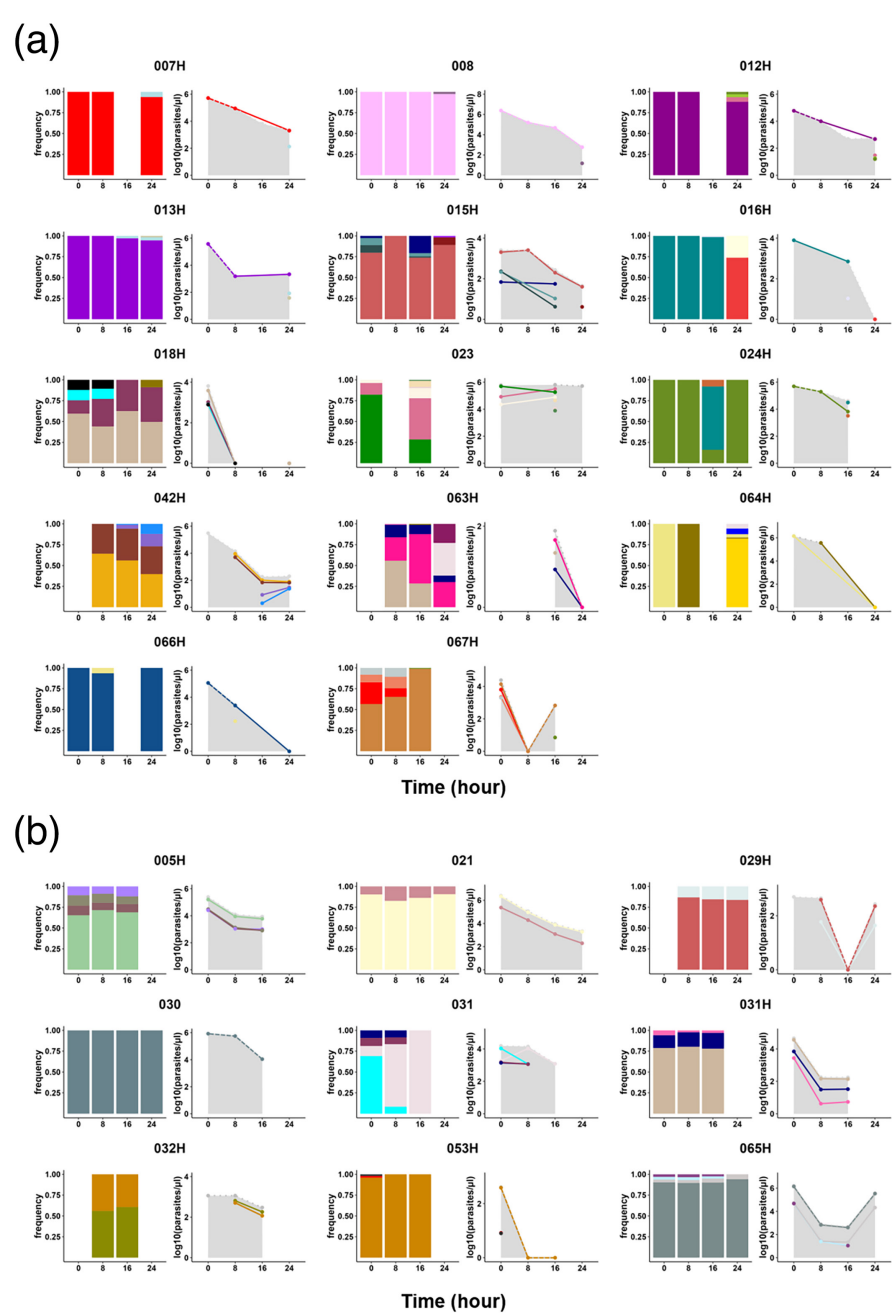
Subpopulation clearance curve during antimalarial treatment, with (a) or without (b) sporadic detection of novel microhaplotypes. The left panel of each plot shows the relative abundance of microhaplotype subpopulations throughout the antimalarial treatment period. The right panel of each plot shows parasite densities of these subpopulations, calculated by multiplying the total parasitaemia by the relative abundance of the microhaplotypes. The clearance curves for the total parasite density are shown as a background surface (in grey). Each colour represents a unique microhaplotype within the population. Sporadic detection is defined as the appearance of a new microhaplotype that was not present at H0.

To measure the complexity of microhaplotypes within each isolate, we measured the Shannon entropy, a metric dependent on the number and proportion of each microhaplotype within an isolate. Despite the overall parasitaemia decreasing by an average of 335-fold over 24 h, the median microhaplotype entropy only slightly decreased between H0 and H8 (*P*=0.0388, Mann–Whitney test) and remained stable afterwards (*P*=0.6093, Kruskal–Wallis test) (Fig. S3). This stable MOI is explained by the detection of novel microhaplotypes that were not in circulation at H0. These ‘sporadic’ microhaplotypes were detected in over 60.9% (14/23) of participants, predominantly occurring from 16 h after the start of treatment (Fig. 7), likely indicating the release of sequestered parasites that were previously undetectable. Additionally, half of the participants with sporadic microhaplotypes (007 h, 008, 012 h, 013 h, 016 h, 024 h, 064 h and 066 h) had monoclonal infections at H0 but displayed polyclonal infections at later time points (Fig. 7a). However, most of the sporadic microhaplotypes were very minor genotypes (Fig. 7a).

We inferred the parasitaemia of each circulating genotype based on the total parasitaemia and the proportion of each microhaplotype. Notably, only one individual (067 h) harbouring sporadic microhaplotypes showed a rebound in microhaplotype parasitaemia, indicating that the rebound in total parasitaemia observed was unrelated to the release of new genotypes in circulation after the start of the treatment.

Taken together, we here showed that, from blood samples taken on arrival at the hospital (H0), it is usually not possible to genotype all *P. falciparum* microhaplotypes from an infection. About two-thirds of the time, one or more distinct strains will appear within 24 h, presumably as a result of the release of sequestered *P. falciparum*.

### Parasite clearance is not correlated with drug resistance allele frequencies

We assessed the population allelic frequencies of resistance markers *crt*, *mdr1*, *kelch13*, *dhfr* and *dhps* over the 24-h follow-up period. Of the 45 individuals for whom baseline drug resistance data was available, 95.6% (43/45) carried the chloroquine-resistant allele (K76T) (Fig. S4A). However, the two individuals showing a mixture of sensitive and resistant alleles came from the group treated with artesunate (Fig. S4A). The frequency of the multidrug resistance gene 1 (*mdr1*) remained stable in the population throughout the 24-h treatment period (Fig. S3B). Only one individual with a k13 mutation (V589I) at the time of enrolment was found in the study population (Fig. S4C). All isolates carried a triple mutation of the *dhfr* gene (N51I, C59R and S108N) (Figure S4D). Similarly, 99% of isolates carried the A437G mutation in the *dhps* gene, with 23.2% and 0% a second mutation (Fig. S4E). Overall, we observed no alteration in genotypes across all analysed resistance markers within the same individual during antimalarial treatment.

## Discussion

In recent years, several approaches have been used to study the intra-host dynamics of polyclonal infections, including WGS, which is the most comprehensive approach for genomic epidemiology, providing a complete picture of genetic variation. However, molecular markers, mainly AmpSeq, which combines several highly polymorphic antigen markers, are now the method of choice for high-resolution detection of MOI [23,36]. We first aim in this study to evaluate different methods to study intra-host dynamics of polyclonal infections during a course of parasite clearance with an antimalarial treatment. Using the WGS data, we observed a robust correlation between the three algorithms (*Fws*, THE REAL McCOIL and RoH) used to determine the clonality of an isolate, in particular higher between *Fws* and RoH with 94.3% consistent results between the two metrics. However, 16.1% of polyclonal isolates according to *Fws* were classified as monoclonal according to THE REAL McCOIL. These data indicate that both the *Fws* and RoH algorithms exhibit greater sensitivity in detecting multiple infections compared to THE REAL McCOIL. Indeed, THE REAL McCOIL is a more conservative approach, which provides estimates that minimize the overestimation of MOI by considering the likelihood of observed genetic variation being due to multiple parasite strains rather than sequencing errors or technical artefacts [25]. In this study, more than 79% of the isolates showed concordant results (either monoclonal or polyclonal) between AmpSeq and Fws or RoH. However, the AmpSeq method was able to detect up to six genotypes in an isolate, indicating greater sensitivity of AmpSeq in detecting multiple genomes in an isolate compared to WGS-based methods. Amplicon sequencing targeting highly polymorphic loci has demonstrated the capability to detect minority haplotypes with frequencies as low as 0.1% [27]. While WGS is powerful for whole-genome insights, its ability to detect minor alleles is often limited by the achievable depth of coverage within the budget constraints. In contrast, AmpSeq focuses on highly polymorphic loci, offering a more cost-effective approach to detecting minority genotypes with greater sensitivity.

Previous studies have demonstrated that plasma can be used to detect and quantify the *Plasmodium* DNA by PCR [21,22]. However, these studies also indicated that quantitative Polymerase Chain Reaction (qPCR) using whole blood DNA exhibits greater sensitivity than qPCR with plasma DNA. In line with the sequestration phenomenon of *P. falciparum* as shown by quantifying the plasma levels of HRP2 or parasite DNA [18,20], we hypothesized that genotyping parasites using DNA extracted from plasma might detect sequestered *P. falciparum* genotypes. This hypothesis assumes that plasma may contain parasite DNA released from prior schizont ruptures of all the parasite genotypes, which could originate from multiple sources: (1) DNA released by merozoites that fail to invade the RBCs, lysing in the extracellular environment, (2) residual DNA within schizonts that is not packaged into merozoites released along with schizont contents during rupture and (3) DNA released by the lysis of infected RBCs, either mediated by the immune system or triggered by antimalarial drug action. This would be unaffected by the parasite sequestration at the time of blood sample collection. However, our findings revealed significantly higher microhaplotype complexity in the RBC pellet compared to plasma. These results indicate that AmpSeq using gDNA from RBC pellets is more sensitive compared to AmpSeq using gDNA from plasma. To what extent the RBC pellet contains DNA from prior schizont ruptures remains to be determined. Consistent with previous findings where low levels of parasitaemia were more effectively detected by qPCR using parasite DNA from whole blood compared to plasma [21], our study also found that parasite load influences the sensitivity of AmpSeq when using plasma as the DNA source. Indeed, the median parasitaemia of isolates with the higher MOI in the RBC pellet compared to plasma was 34.4-fold lower compared to the median parasitaemia of isolates with the same MOI in both biological materials. Interestingly, at parasitaemia levels exceeding 1,000 parasites per microlitre, the same MOI was detected in both plasma and pellet samples in 85.4% of the isolates. This indicates that plasma can be effectively used for parasite genotyping in retrospective studies.

We observed high variability in parasite clearance half-life within both treatment groups, with faster parasite clearance rate in children treated with artesunate compared to those treated with quinine. Artesunate is a fast-acting antimalarial drug with a pronounced effect on ring-stage parasites, whereas quinine primarily targets mature trophozoites [37]. Rapid clearance results from artesunate action on the circulating ring-stage parasites and their subsequent removal predominantly by the spleen, preventing parasite sequestration [38,39]. In line with our findings, a recent study of Ghanaian children showed a prolonged delay in parasite clearance among children with severe malaria, even after 3 days of treatment with artesunate [40]. This increased time to parasite clearance was associated with age, low haemoglobin levels and a high number of previous malaria diagnoses [40]. Other studies have noted variations in parasite clearance rates in children treated for uncomplicated falciparum malaria with artemisinin-based combination therapy, both in Kenya and Tanzania [41,42]. These findings suggest that factors other than drug resistance may contribute to slower parasite clearance in certain infections, as there is no evidence of artemisinin resistance in these regions. It has been shown that, in areas where artemisinin resistance is not present, the parasite clearance time can be affected by factors such as the age of the host, host-acquired immunity, initial parasitaemia levels and the developmental stages of the parasite [11,13]. Furthermore, although controversial, studies have shown a link between the genetic complexity of * P. falciparum* and delayed parasite clearance in Africa [14,15]. Our data show that the *P. falciparum* clearance rate in children with severe malaria is independent of parasite MOI at the onset of treatment. Notably, *P. falciparum* clearance was faster in polyclonal infections in quinine-treated children. However, this difference was attributed to significantly higher parasitaemia at enrolment in individuals treated with quinine.

Interestingly, during antimalarial treatment, a high rate of ‘rebound’ in total parasitaemia and specific parasite subpopulations of parasitaemia was observed. Specifically, an increase in total parasitaemia between two time points was detected in 34.8% of individuals. In four individuals (023, 031, 063 h and 064 h), minor genotypes present at baseline (H0) increased in relative frequency, becoming the dominant microhaplotype. Additionally, sporadic detection of microhaplotypes occurred in over 60.9% of participants, predominantly from 16 h after treatment initiation. Half of the participants with sporadic microhaplotypes had monoclonal infections at baseline but exhibited polyclonal infections at subsequent time points. These findings potentially highlight the release of sequestered *P. falciparum* genotypes during antimalarial treatment [18]. Interestingly, none of these sporadic microhaplotypes were detected in pellet or plasma samples at H0, despite the much higher parasite density at that time point that would have facilitated the detection of a rare genotype. Either these microhaplotypes recently emerged from the liver, with no or little parasite DNA in the plasma yet, or, probably more likely, these sporadic microhaplotypes are present at extremely low parasitaemia, making them undetectable in plasma, and were all sequestered at H0. These findings are consistent with previous studies using *msp1* and *msp2* genotyping, which reported that 30% of children acquired an additional genotype within 24 h after initiating treatment for uncomplicated infections [43]. This underscores the limitations of relying on a single pre-treatment sample for MOI determination, particularly in clinical efficacy trials that depend on PCR-corrected outcomes. Additional sampling within the first 24 h is necessary to capture the full diversity of infections and improve the accuracy of genotyping results.

Additionally, a study by Marks et al. [44] reported a parasitological rebound effect in infants treated with a single dose of sulphadoxine-pyrimethamine, attributed to the selection of drug-resistant parasites shortly after drug clearance [44]. However, no mutation associated with artemisinin resistance has yet been identified in Benin [9,10]. In addition, no alterations were observed in allele frequencies across all analysed resistance markers within the same individual post-antimalarial treatment. Two other studies using AmpSeq detected sporadic microhaplotypes or an increase in the frequency of minor microhaplotypes during the treatment of uncomplicated *P. falciparum* infections in regions with no signs of artemisinin resistance [41,42]. In falciparum malaria, parasitized red blood cells circulate in the peripheral blood for only one-third of the 48 h asexual cycle [19]; for the remainder of the cycle, they are sequestered in the venules and capillaries. For these infections, the peripheral blood parasitaemia at a given time point does not reflect the total parasite burden.

Limitations of this study include the lack of sampling time points beyond 24 h post-treatment, which affected the accurate determination of parasite clearance half-life for some individuals using the standard WWARN method [45]. This was due to the fact that the parasitaemia reduction rate was less than 50% of the initial parasitaemia at the last recorded time point. Therefore, we used linear interpolation to estimate the time required for a 50% reduction in parasitaemia, referred to as the extrapolated parasite clearance half-life (PC_50%_). However, unlike therapeutic efficacy studies typically focusing on day 3, 7 and 21/28, we were able to monitor the dynamics of *P. falciparum* complexity during the initial management of severe malaria. Additionally, the 8-h sampling intervals could potentially miss finer dynamics of parasite clearance, particularly rapid fluctuations in parasite densities that occur shortly after drug administration. While these intervals were selected to balance logistical feasibility and patient safety, denser sampling could provide a more precise determination of parasite clearance dynamics.

This study demonstrates that combining different haplotyping techniques can effectively determine genetic complexity. However, AmpSeq is the most sensitive approach and should be the first choice for genotyping to monitor the evolution of genotype clearance following antimalarial treatment. Additionally, plasma can be effectively used for parasite genotyping at sufficient parasitaemia levels. We demonstrated that genotyping a blood sample from a patient on arrival at the hospital is typically not sufficient to detect all parasite genotypes present in the infection.

## Supplementary material

10.1099/mgen.0.001386 Uncited Supplementary Material 1.

10.1099/mgen.0.001386 Uncited Supplementary Material 2.

## Abbreviations

ACTs: artemisinin-based combination therapies
AmpSeq: amplicon sequencing
CM: cerebral malaria
*Fws*: fraction of within-sample
gDNA: genomic DNA
MalariaGEN: Malaria Genomic Epidemiology Network
MOI: multiplicity of infection
RBCs: red blood cells
RoH: runs of homozygosity
SM: severe malaria
SMA: severe malaria anaemia
SNCM: severe non-cerebral malaria
VCF: variant calling files
WBCs: white blood cells
WGS: whole-genome sequencing
WHO: World Health Organization
