# Supplementary material for: Detection of novel Plasmodium falciparum haplotypes under treatment pressure in paediatric severe malaria
Source: Microb Genom. 2025 May 9;11(5):001386. doi: 10.1099/mgen.0.001386 (PMC12064853; doi:10.1099/mgen.0.001386)
Supplement: Uncited Supplementary Material 1. [file mgen-11-01386-s001.pdf]

Supplementary Material

Detection of novel *Plasmodium falciparum* haplotypes under treatment pressure in pediatric severe malaria

Balotin Fogang[1], Emilie Guillochon[2], Claire Kamaliddin[2&], Gino Agbota [2,3], Sem Ezinmegnon [2,3], Maroufou Jules Alao[4], Philippe Deloron[2], Gwladys Bertin[2], Antoine Claessens[1*]

1 LPHI, CNRS, INSERM, University of Montpellier, Montpellier, France

2 Université Paris Cité, MERIT, IRD, Paris, France

3 Institut de Recherche Clinique du Bénin (IRCB), Abomey-Calavi, Benin

4 Paediatric Department, Mother and Child University and Hospital Center (CHU-MEL), Cotonou, Benin

& Current affiliation: Cumming School of Medicine, The University of Calgary, Calgary, Alberta, Canada

*Correspondence antoine.claessens@umontpellier.fr

Repositories. All WGS sequencing data generated and analyzed in this study have been deposited in the European Nucleotide Archive (ENA) under the study accession number PRJEB2136 for public access. The raw reads of the AmpSeq data have also been made publicly available on Zenodo under the accession number 10.5281/zenodo.13224728. Epidemiological data of study participants and Amplicon Sequencing results are provided as supplementary tables (Supplementary Table 1 and Supplementary Table 2, respectively).

Keywords. *Plasmodium falciparum*, genetic complexity, parasite clearance, severe malaria

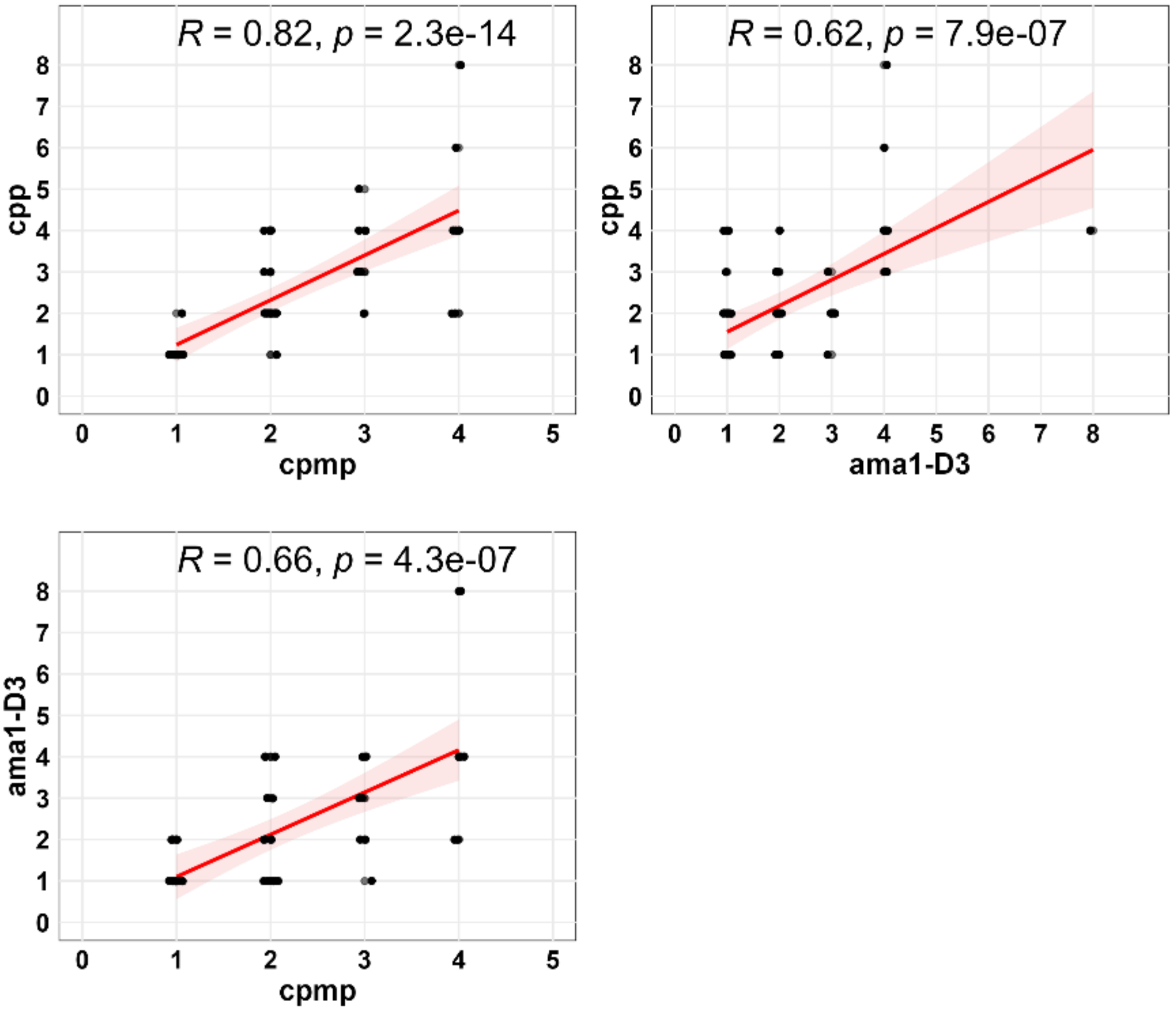

Supplementary Figure 1. Correlation in MOI between AmpSeq makers (cpmp, cpp and ama1-D3).

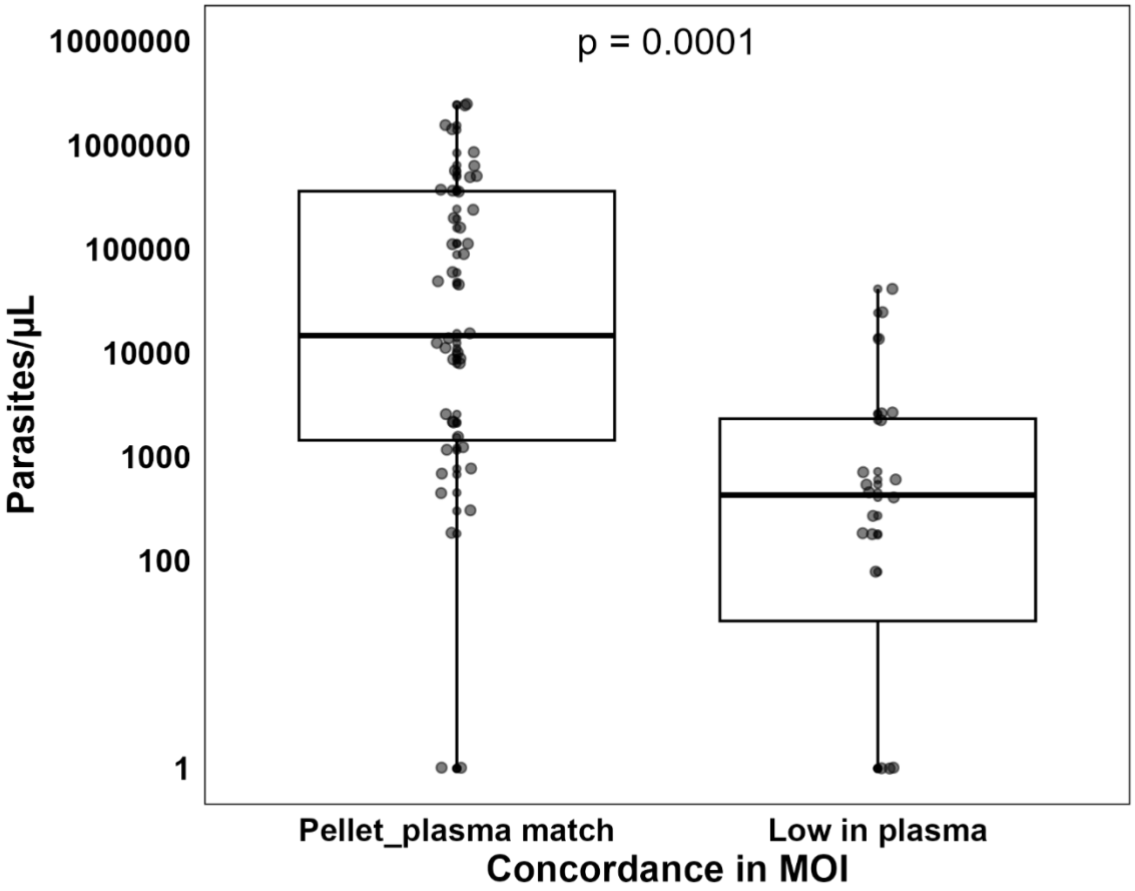

Supplementary Figure 2. Parasitaemia according to the concordance between MOI from paired RBC pellet and plasma samples, determined by AmpSeq.

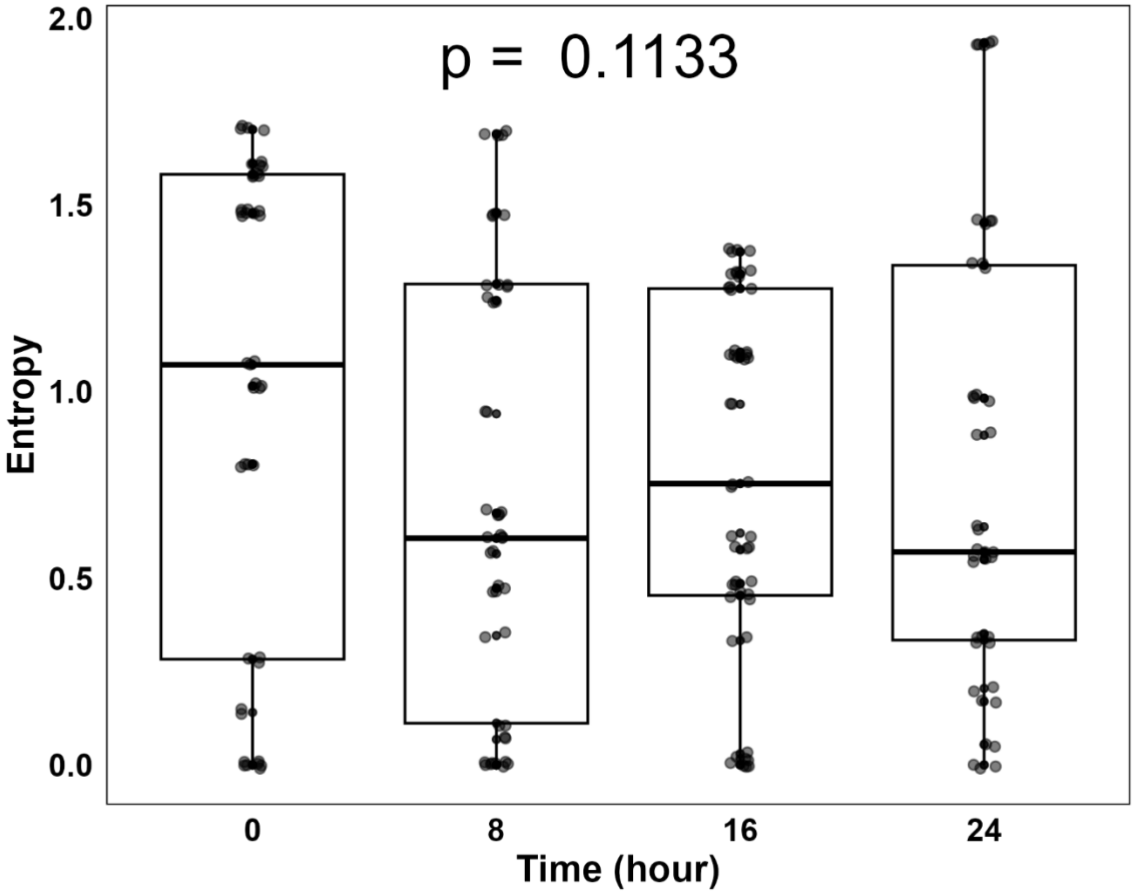

Supplementary Figure 3. Microhaplotype entropy over time during antimalarial treatment.

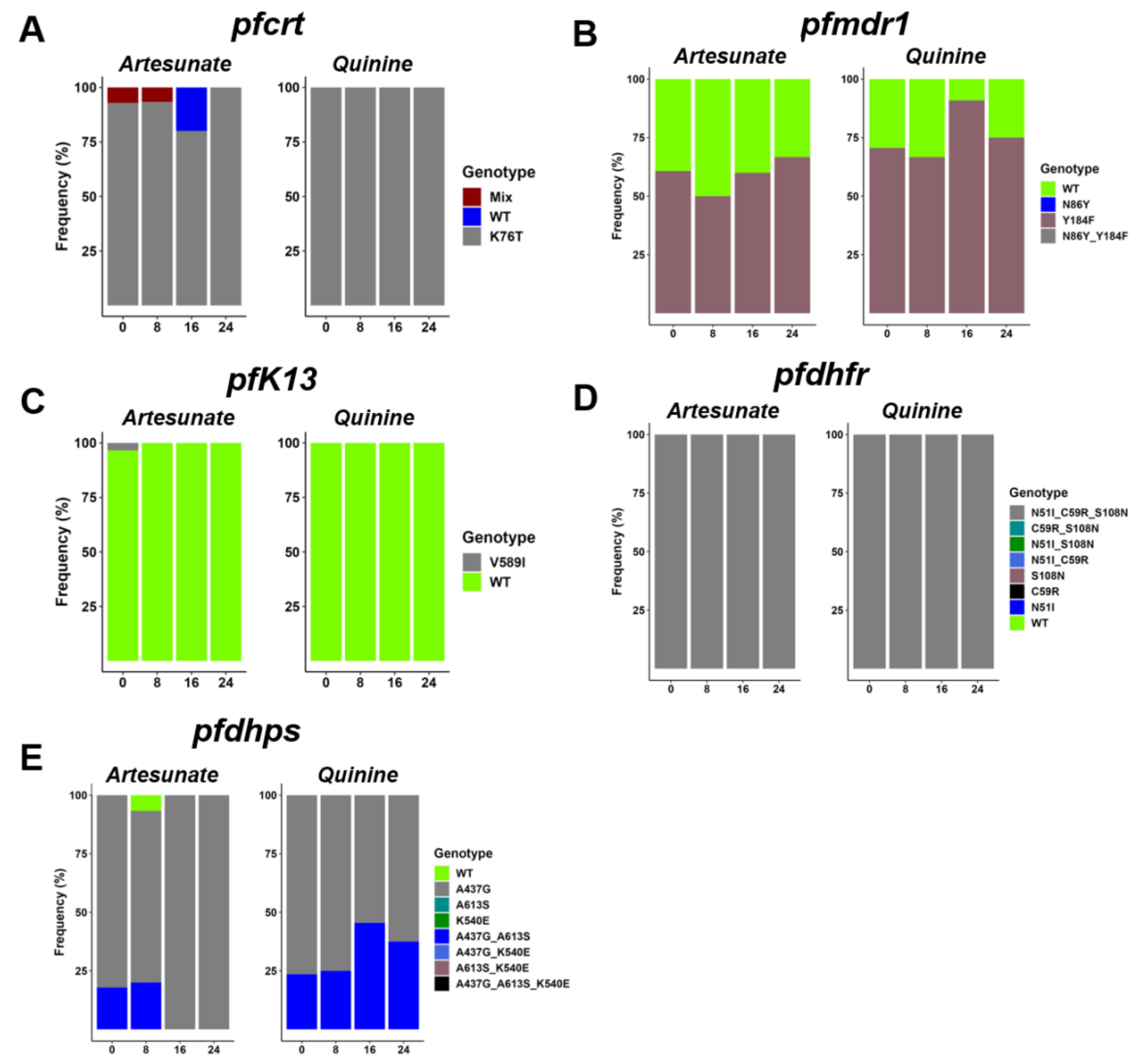

Supplementary Figure 4. Frequency of antimalarial drug resistance markers throughout treatment.

Supplementary Table 2. PCR primers sequences and amplification conditions.

**Primers**	**Amplification Conditions**
**Primary PCR**	**Primary PCR**
cpmp_prim_fw: CGATACAGGACATATAGA<br>cpmp_prim_rv: TTCAATAACATTTACTAGG<br>cpp_prim_fw: TGTCTGAACCAAATTCAA<br>cpp_prim_rv: GAATTTGTCACATTTGATGA<br>ama1-D3_prim_fw : GTTTAATTAACAATTCATCATAC<br>ama1-D3_prim_rv: GTGTTGTATGTGATGCTC	95°C for 3′, 20 cycles of 98°C for 20″ and 54°C for 15″ and 72°C for 45″, followed by 72°C for 2′
**Nested PCR**	**Nested PCR**
cpmp_nested: CATAAGTCATTAAAATTTATGGAT<br>cpmp_nested: CGTTACTATCAAGATCGTTAATAT<br>cpp_nested: CAAGTTCACTTTTGGGAAATG<br>cpp_nested: ATTACTACCTTTCAGCATATCCGA<br>ama1-D3_ nested: TACTACTGCTTTGTCCCATC<br>ama1-D3_ nested: TCAGGATCTAACATTTCATC	95°C for 3′, 10 cycles of 98°C for 20″ and 55 °C for 15″ and 72 °C for 45″, 10 cycles of 98 °C for 20″ and 68°C for 15″ and 72 °C for 45″followed by 72 °C for 1′30″

fw: Forward. rv: Reverse.

Assessment of amplicon sequencing quality using PhiX Spike-In Controls

The quality of the sequencing run was assessed by investigating the sequencing error rate using PhiX spike-in controls, which demonstrated error rates of 1.8% for Read 1 and 2.06% for Read 4 (Supplementary Table 4). Additionally, the sequencing run produced high-quality data, with over 90% of bases at or above Q30 for most reads.

Supplementary Table 4. Summary Metrics of Sequencing Run Performance for Indexed and Non-Indexed Reads.

Level	Yield	Projected Yield	Aligned	Error Rate	Intensity C1	%>=Q30
Read 1	5.68	5.68	7.34	1.80	223	93.80
Read 2 (I)	0.15	0.15	0.00	nan	456	90.80
Read 3 (I)	0.15	0.15	0.00	nan	419	96.38
Read 4	5.68	5.68	7.29	2.06	205	86.60
Non-indexed	11.36	11.36	7.31	1.93	214	90.20
Total	11.66	11.66	7.31	1.93	326	90.29

Read 1 and Read 4 correspond to PhiX spike-in controls. Read 2 and 3 correspond to the sequencing index reads. Non-indexed correspond to combined yield of Read 1 and Read 4.

Appendix 1: Determining the Multiplicity of Infection (MOI) based on Whole Genome Sequencing (WGS)

To assess the MOI from the 192 good-quality genomes, three algorithms were tested: F_{WS}, THE REAL McCOIL and Runs of Homozygosity (RoH). Any genome with $F_{WS} > 0.95$ was considered monoclonal. The RoH method categorises isolates as monoclonal (a single dominant genotype), biclonal (2 dominant genotypes) or multiclonal (3+ dominant genotypes) (Figure B), as defined in Pearson et al [1]. THE REAL McCOIL method estimates the actual number of genotypes present in an isolate. We observed a robust correlation between the three methods, particularly higher *Fws* and RoH ($r= 0.81$, $p < 0.0001$) (Figure A). In addition, 94.3% (181/192) of isolates showed consistent results (either monoclonal or polyclonal) between *Fws* and RoH (Figure C). However, 83.9% (161/192) of the isolates showed consistent results

(either monoclonal or polyclonal) for both *Fws* and THE REAL McCOIL, while 16.1% (31/192) were polyclonal according to F_{WS} but monoclonal according to THE REAL McCOIL, making the latter algorithm more conservative (Figure C). These data indicate that both the *Fws* and RoH algorithms exhibit greater sensitivity in detecting multiple infections compared to THE REAL McCOIL. By WGS-based methods, 52.3% (34/65) of the infections were polyclonal for at least one timepoint over the 24 hours follow-up.

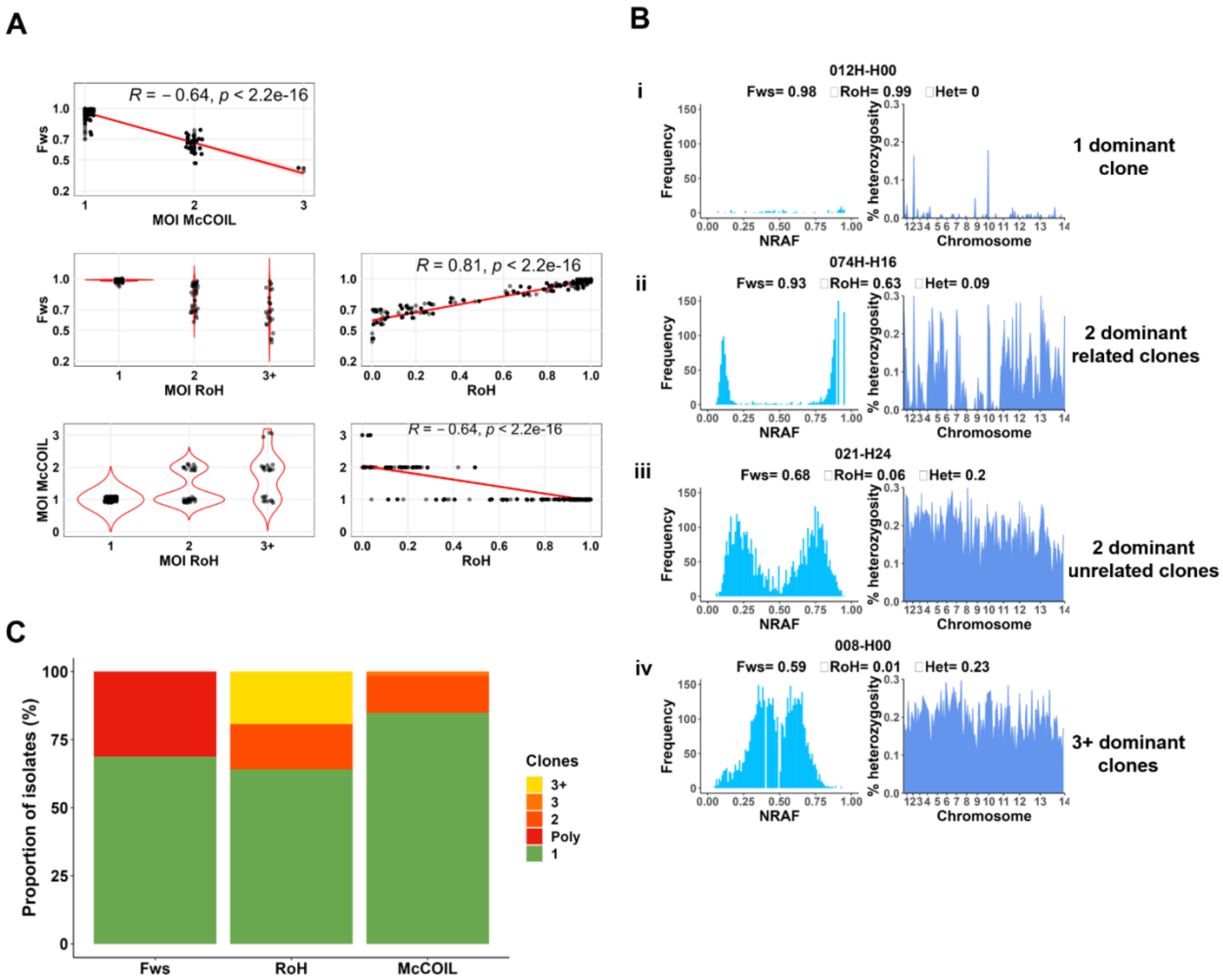

Figure. Comparison of different approaches to determine the multiplicity of the infection. A) Correlation between three different WGS-based algorithms (Fws, McCOIL, RoH) used to determine the MOI from 192 genomes. B) Four illustrative samples showing the non-reference allele frequency (NRAF) distribution across all heterozygous SNPs (left) and heterozygosity

calculated in 100-kb bins (right) to highlight RoH), as defined in Pearson et al [1]. Sample i is clonal, with within-sample heterozygosity (Het) of 0 and Fws at 0.98. Sample ii and iii are likely two genotypes, as evidenced by the bimodal distribution of NRAF, with the apex each peak indicating the relative proportion of each genotype within the sample. However only sample ii is showing runs of homozygosity (RoH), indicating that the 2 genotypes within that sample are genetically related. Sample iv contains a complete mixture of genotypes,. All genomes are available in Supplementary Data 1. C) Clonality levels according to WGS-based algorithms.

Supplementary Data 1. Non-reference allele frequency (NRAF) distribution across all heterozygous SNPs (left) and heterozygosity calculated in 100-kb bins (right) to highlight RoH.

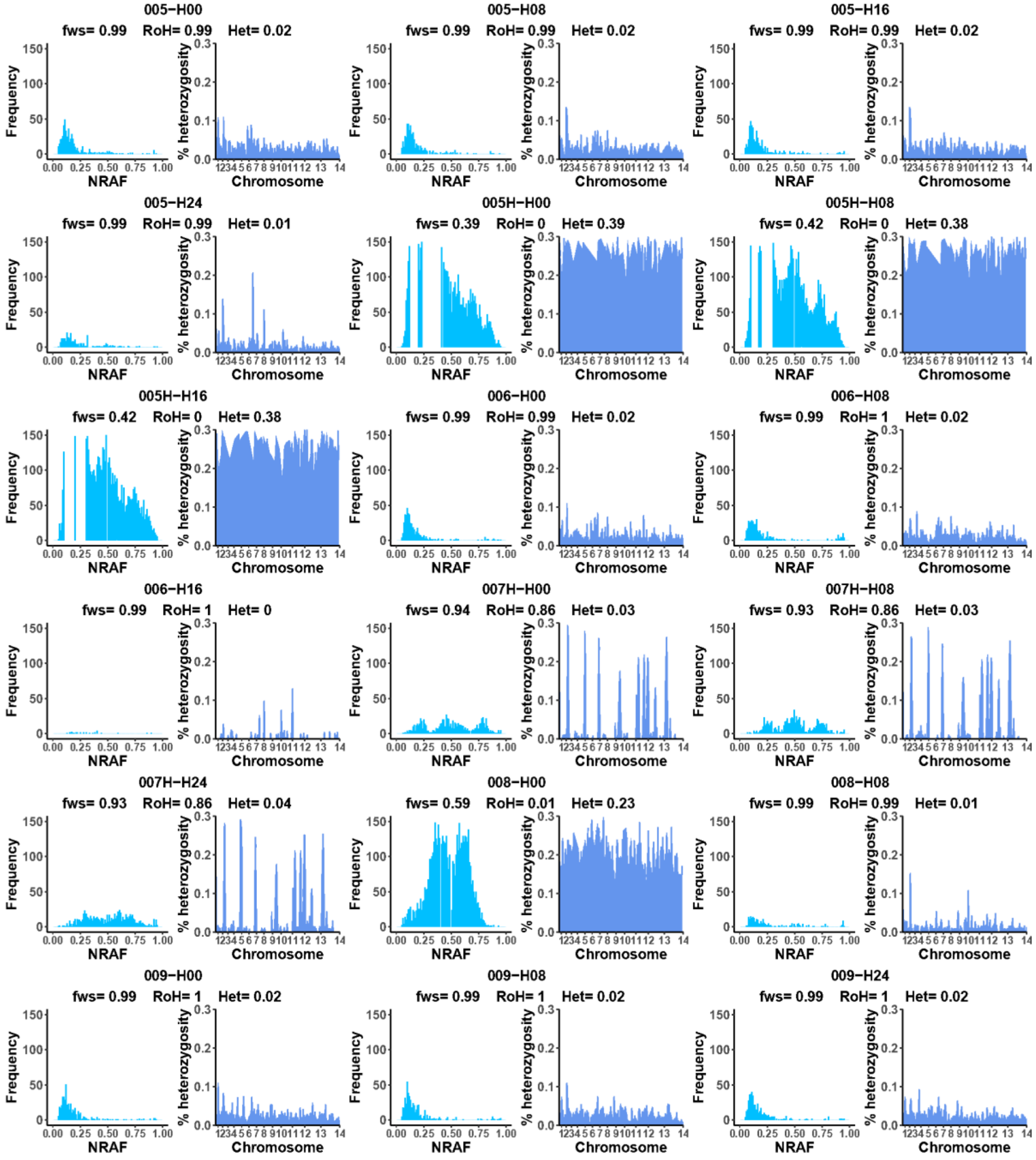
005−H00
fws= 0.99 RoH= 0.99 Het= 0.02
005−H08
fws= 0.99 RoH= 0.99 Het= 0.02
005−H16
fws= 0.99 RoH= 0.99 Het= 0.02
005−H24
fws= 0.99 RoH= 0.99 Het= 0.01
005H−H00
fws= 0.39 RoH= 0 Het= 0.39
005H−H08
fws= 0.42 RoH= 0 Het= 0.38
005H−H16
fws= 0.42 RoH= 0 Het= 0.38
006−H00
fws= 0.99 RoH= 0.99 Het= 0.02
006−H08
fws= 0.99 RoH= 1 Het= 0.02
006−H16
fws= 0.99 RoH= 1 Het= 0
007H−H00
fws= 0.94 RoH= 0.86 Het= 0.03
007H−H08
fws= 0.93 RoH= 0.86 Het= 0.03
007H−H24
fws= 0.93 RoH= 0.86 Het= 0.04
008−H00
fws= 0.59 RoH= 0.01 Het= 0.23
008−H08
fws= 0.99 RoH= 0.99 Het= 0.01
009−H00
fws= 0.99 RoH= 1 Het= 0.02
009−H08
fws= 0.99 RoH= 1 Het= 0.02
009−H24
fws= 0.99 RoH= 1 Het= 0.02
Frequency
NRAF
% heterozygosity
Chromosome

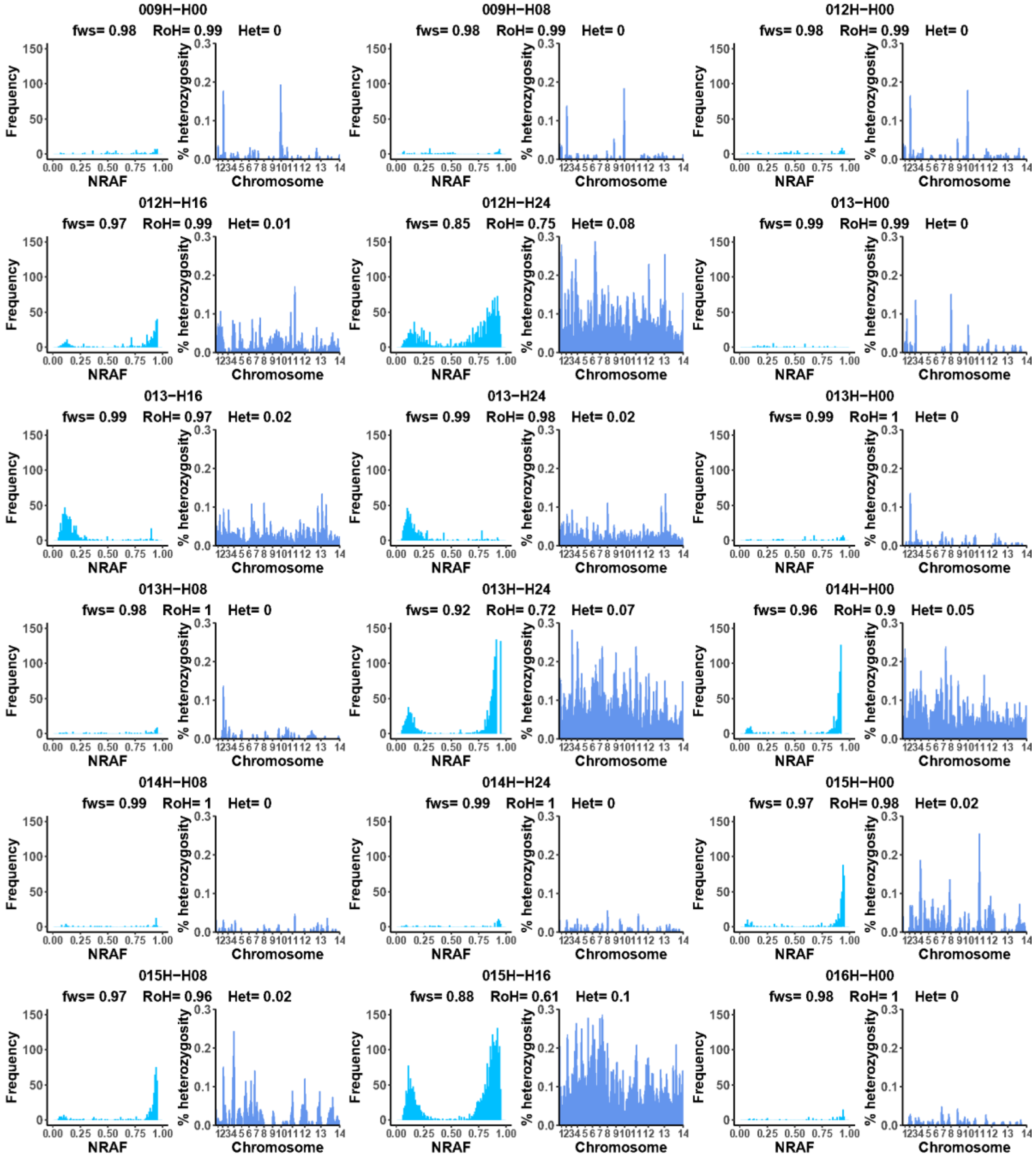

009H−H00
fws= 0.98 RoH= 0.99 Het= 0
009H−H08
fws= 0.98 RoH= 0.99 Het= 0
012H−H00
fws= 0.98 RoH= 0.99 Het= 0
012H−H16
fws= 0.97 RoH= 0.99 Het= 0.01
012H−H24
fws= 0.85 RoH= 0.75 Het= 0.08
013−H00
fws= 0.99 RoH= 0.99 Het= 0
013−H16
fws= 0.99 RoH= 0.97 Het= 0.02
013−H24
fws= 0.99 RoH= 0.98 Het= 0.02
013H−H00
fws= 0.99 RoH= 1 Het= 0
013H−H08
fws= 0.98 RoH= 1 Het= 0
013H−H24
fws= 0.92 RoH= 0.72 Het= 0.07
014H−H00
fws= 0.96 RoH= 0.9 Het= 0.05
014H−H08
fws= 0.99 RoH= 1 Het= 0
014H−H24
fws= 0.99 RoH= 1 Het= 0
015H−H00
fws= 0.97 RoH= 0.98 Het= 0.02
015H−H08
fws= 0.97 RoH= 0.96 Het= 0.02
015H−H16
fws= 0.88 RoH= 0.61 Het= 0.1
016H−H00
fws= 0.98 RoH= 1 Het= 0
Frequency
NRAF
% heterozygosity
Chromosome

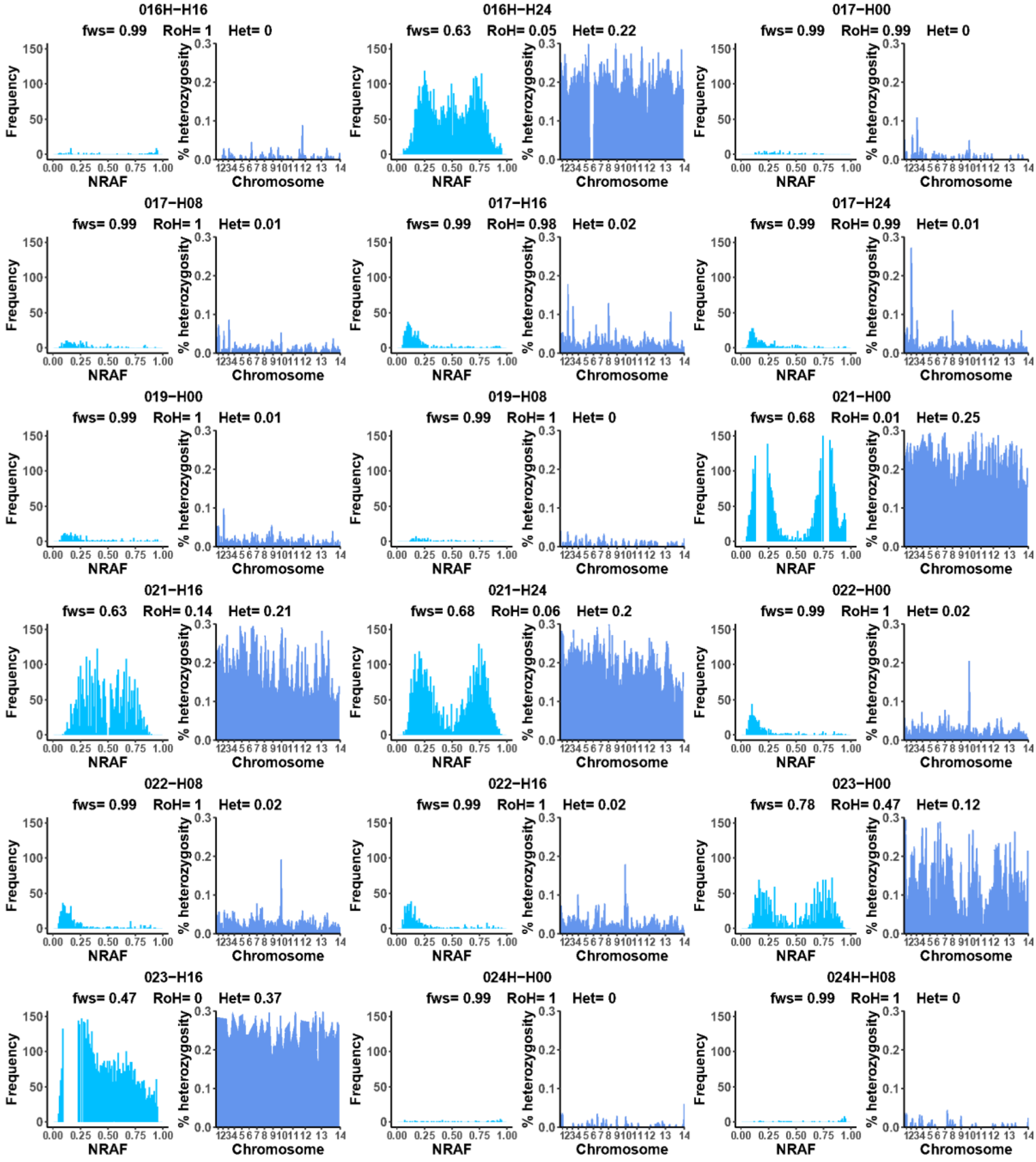
016H−H16
fws= 0.99 RoH= 1 Het= 0
016H−H24
fws= 0.63 RoH= 0.05 Het= 0.22
017−H00
fws= 0.99 RoH= 0.99 Het= 0
017−H08
fws= 0.99 RoH= 1 Het= 0.01
017−H16
fws= 0.99 RoH= 0.98 Het= 0.02
017−H24
fws= 0.99 RoH= 0.99 Het= 0.01
019−H00
fws= 0.99 RoH= 1 Het= 0.01
019−H08
fws= 0.99 RoH= 1 Het= 0
021−H00
fws= 0.68 RoH= 0.01 Het= 0.25
021−H16
fws= 0.63 RoH= 0.14 Het= 0.21
021−H24
fws= 0.68 RoH= 0.06 Het= 0.2
022−H00
fws= 0.99 RoH= 1 Het= 0.02
022−H08
fws= 0.99 RoH= 1 Het= 0.02
022−H16
fws= 0.99 RoH= 1 Het= 0.02
023−H00
fws= 0.78 RoH= 0.47 Het= 0.12
023−H16
fws= 0.47 RoH= 0 Het= 0.37
024H−H00
fws= 0.99 RoH= 1 Het= 0
024H−H08
fws= 0.99 RoH= 1 Het= 0
Frequency
NRAF
% heterozygosity
Chromosome

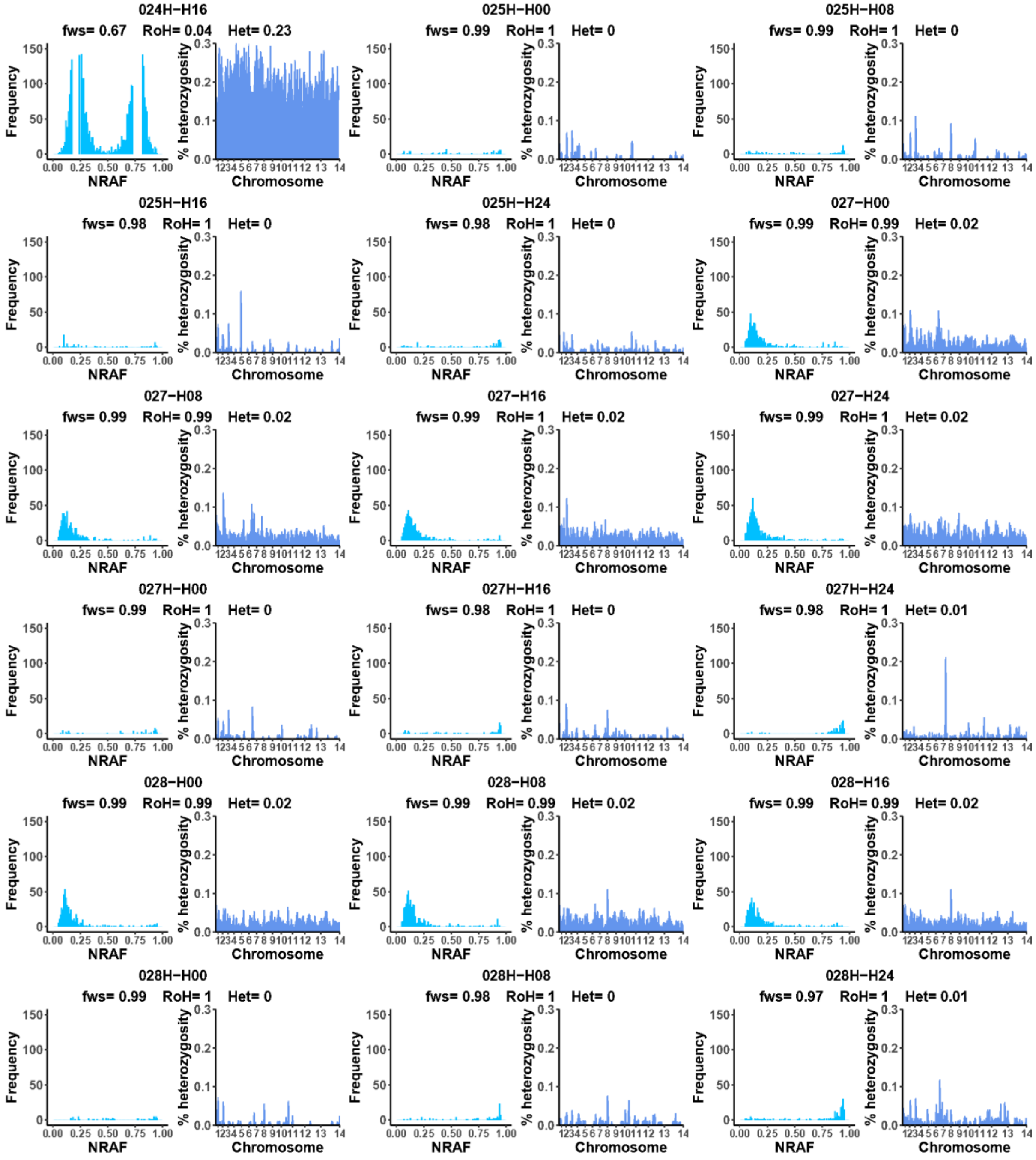
024H−H16
fws= 0.67 RoH= 0.04 Het= 0.23
025H−H00
fws= 0.99 RoH= 1 Het= 0
025H−H08
fws= 0.99 RoH= 1 Het= 0
025H−H16
fws= 0.98 RoH= 1 Het= 0
025H−H24
fws= 0.98 RoH= 1 Het= 0
027−H00
fws= 0.99 RoH= 0.99 Het= 0.02
027−H08
fws= 0.99 RoH= 0.99 Het= 0.02
027−H16
fws= 0.99 RoH= 1 Het= 0.02
027−H24
fws= 0.99 RoH= 1 Het= 0.02
027H−H00
fws= 0.99 RoH= 1 Het= 0
027H−H16
fws= 0.98 RoH= 1 Het= 0
027H−H24
fws= 0.98 RoH= 1 Het= 0.01
028−H00
fws= 0.99 RoH= 0.99 Het= 0.02
028−H08
fws= 0.99 RoH= 0.99 Het= 0.02
028−H16
fws= 0.99 RoH= 0.99 Het= 0.02
028H−H00
fws= 0.99 RoH= 1 Het= 0
028H−H08
fws= 0.98 RoH= 1 Het= 0
028H−H24
fws= 0.97 RoH= 1 Het= 0.01
Frequency
NRAF
% heterozygosity
Chromosome

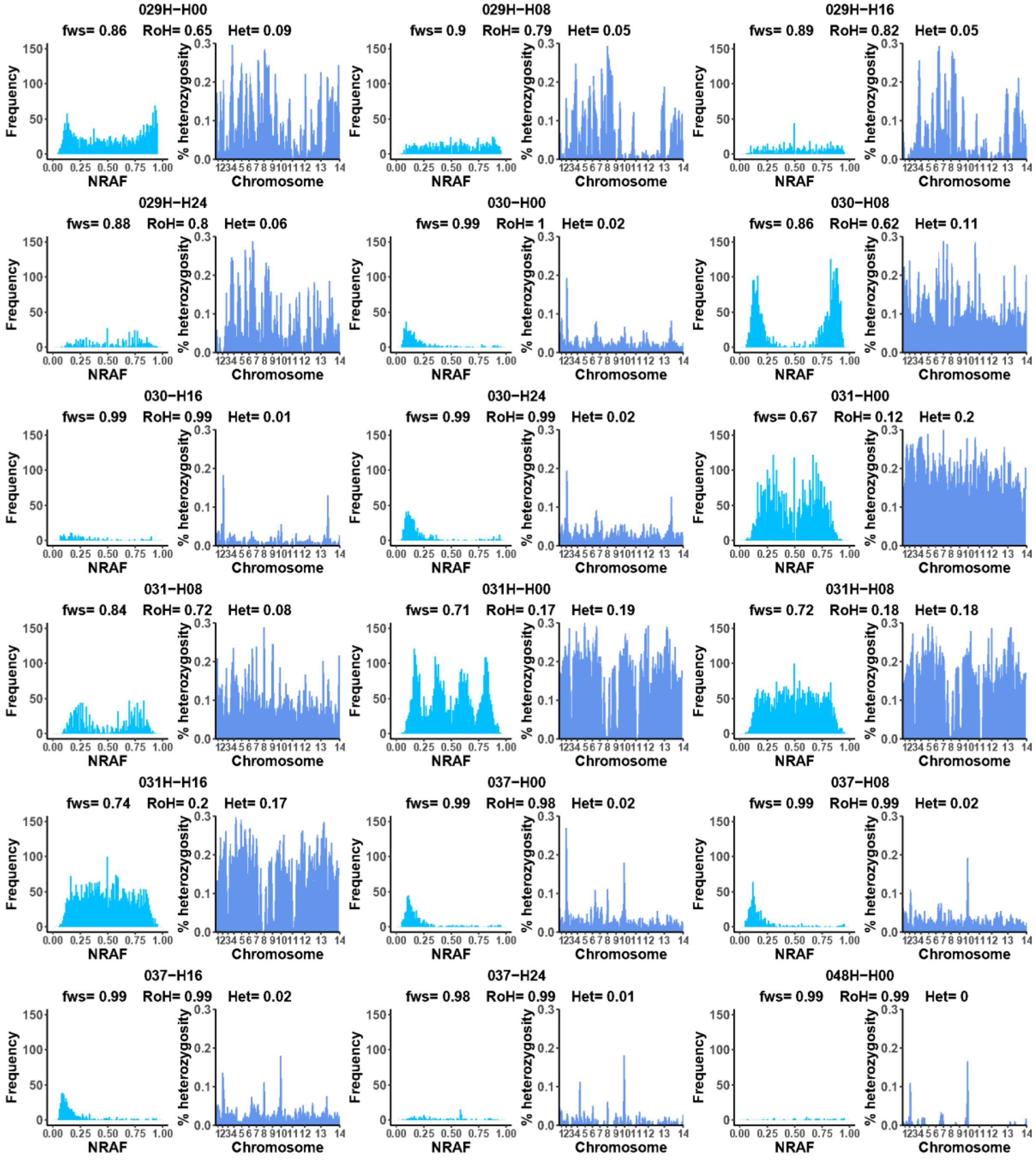
029H−H00
fws= 0.86 RoH= 0.65 Het= 0.09
029H−H08
fws= 0.9 RoH= 0.79 Het= 0.05
029H−H16
fws= 0.89 RoH= 0.82 Het= 0.05
029H−H24
fws= 0.88 RoH= 0.8 Het= 0.06
030−H00
fws= 0.99 RoH= 1 Het= 0.02
030−H08
fws= 0.86 RoH= 0.62 Het= 0.11
030−H16
fws= 0.99 RoH= 0.99 Het= 0.01
030−H24
fws= 0.99 RoH= 0.99 Het= 0.02
031−H00
fws= 0.67 RoH= 0.12 Het= 0.2
031−H08
fws= 0.84 RoH= 0.72 Het= 0.08
031H−H00
fws= 0.71 RoH= 0.17 Het= 0.19
031H−H08
fws= 0.72 RoH= 0.18 Het= 0.18
031H−H16
fws= 0.74 RoH= 0.2 Het= 0.17
037−H00
fws= 0.99 RoH= 0.98 Het= 0.02
037−H08
fws= 0.99 RoH= 0.99 Het= 0.02
037−H16
fws= 0.99 RoH= 0.99 Het= 0.02
037−H24
fws= 0.98 RoH= 0.99 Het= 0.01
048H−H00
fws= 0.99 RoH= 0.99 Het= 0
Frequency
NRAF
% heterozygosity
Chromosome

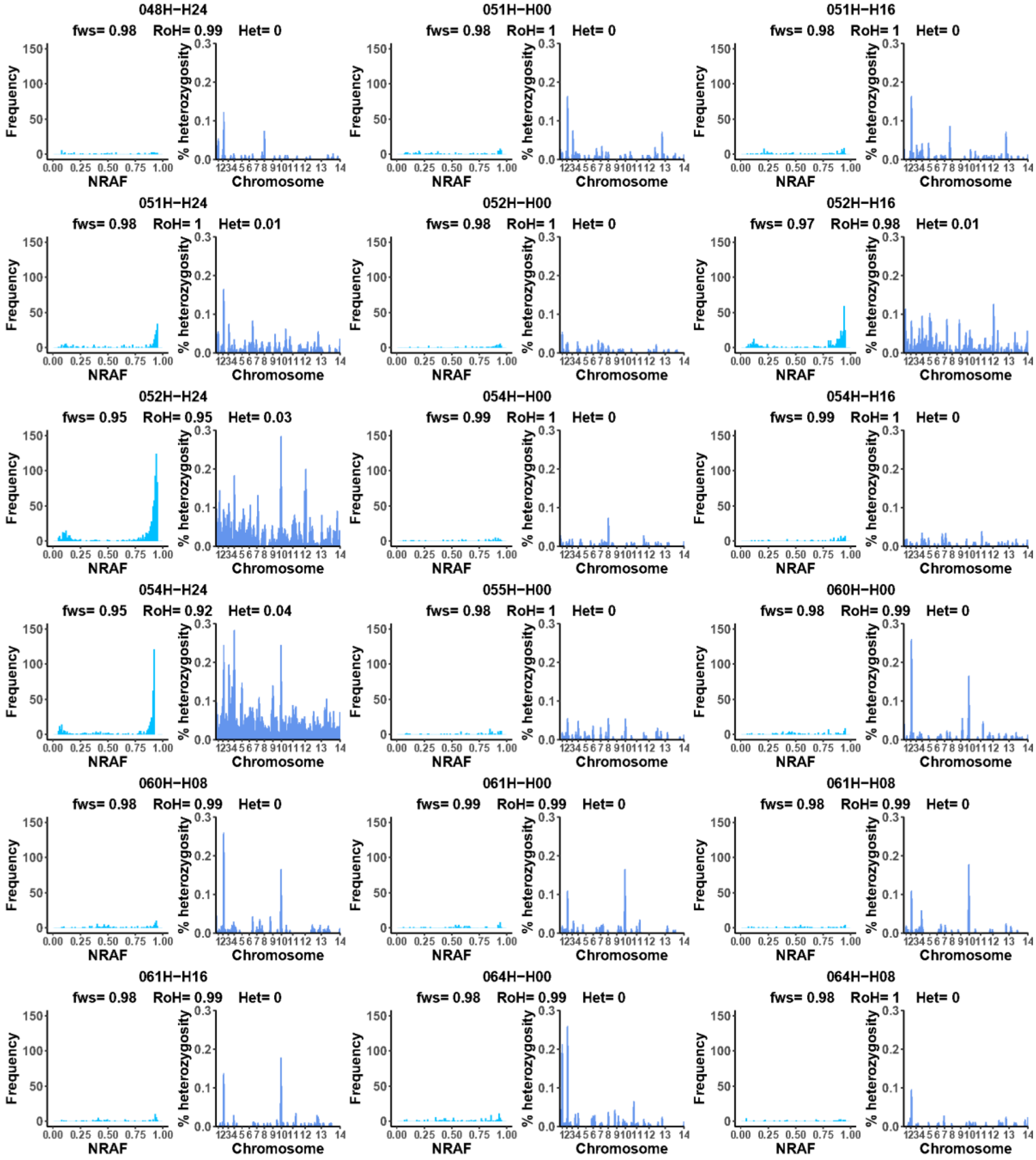
048H−H24
fws= 0.98 RoH= 0.99 Het= 0
051H−H00
fws= 0.98 RoH= 1 Het= 0
051H−H16
fws= 0.98 RoH= 1 Het= 0
051H−H24
fws= 0.98 RoH= 1 Het= 0.01
052H−H00
fws= 0.98 RoH= 1 Het= 0
052H−H16
fws= 0.97 RoH= 0.98 Het= 0.01
052H−H24
fws= 0.95 RoH= 0.95 Het= 0.03
054H−H00
fws= 0.99 RoH= 1 Het= 0
054H−H16
fws= 0.99 RoH= 1 Het= 0
054H−H24
fws= 0.95 RoH= 0.92 Het= 0.04
055H−H00
fws= 0.98 RoH= 1 Het= 0
060H−H00
fws= 0.98 RoH= 0.99 Het= 0
060H−H08
fws= 0.98 RoH= 0.99 Het= 0
061H−H00
fws= 0.99 RoH= 0.99 Het= 0
061H−H08
fws= 0.98 RoH= 0.99 Het= 0
061H−H16
fws= 0.98 RoH= 0.99 Het= 0
064H−H00
fws= 0.98 RoH= 0.99 Het= 0
064H−H08
fws= 0.98 RoH= 1 Het= 0
Frequency
NRAF
% heterozygosity
Chromosome

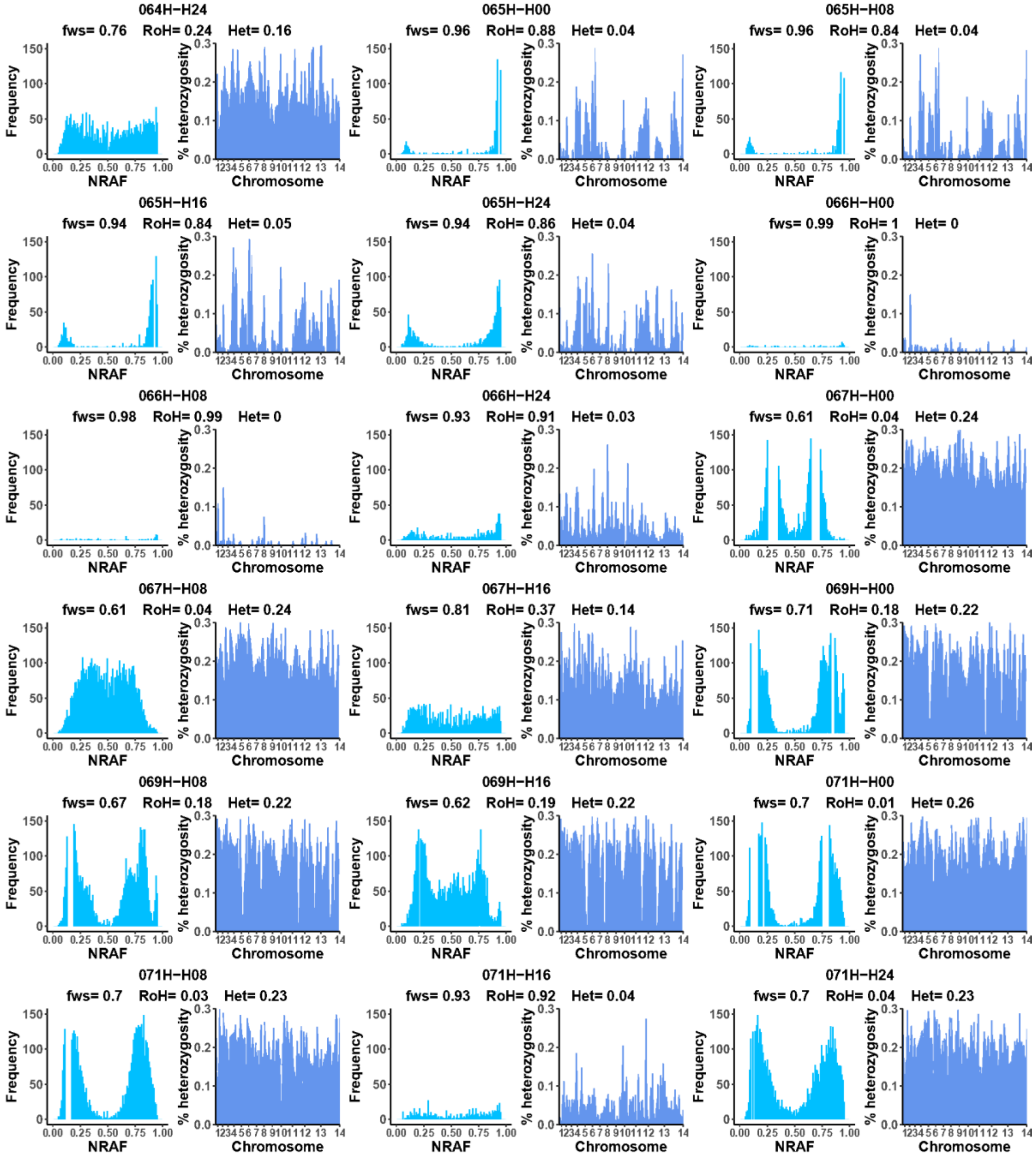
064H−H24
fws= 0.76 RoH= 0.24 Het= 0.16
065H−H00
fws= 0.96 RoH= 0.88 Het= 0.04
065H−H08
fws= 0.96 RoH= 0.84 Het= 0.04
065H−H16
fws= 0.94 RoH= 0.84 Het= 0.05
065H−H24
fws= 0.94 RoH= 0.86 Het= 0.04
066H−H00
fws= 0.99 RoH= 1 Het= 0
066H−H08
fws= 0.98 RoH= 0.99 Het= 0
066H−H24
fws= 0.93 RoH= 0.91 Het= 0.03
067H−H00
fws= 0.61 RoH= 0.04 Het= 0.24
067H−H08
fws= 0.61 RoH= 0.04 Het= 0.24
067H−H16
fws= 0.81 RoH= 0.37 Het= 0.14
069H−H00
fws= 0.71 RoH= 0.18 Het= 0.22
069H−H08
fws= 0.67 RoH= 0.18 Het= 0.22
069H−H16
fws= 0.62 RoH= 0.19 Het= 0.22
071H−H00
fws= 0.7 RoH= 0.01 Het= 0.26
071H−H08
fws= 0.7 RoH= 0.03 Het= 0.23
071H−H16
fws= 0.93 RoH= 0.92 Het= 0.04
071H−H24
fws= 0.7 RoH= 0.04 Het= 0.23
Frequency
NRAF
% heterozygosity
Chromosome

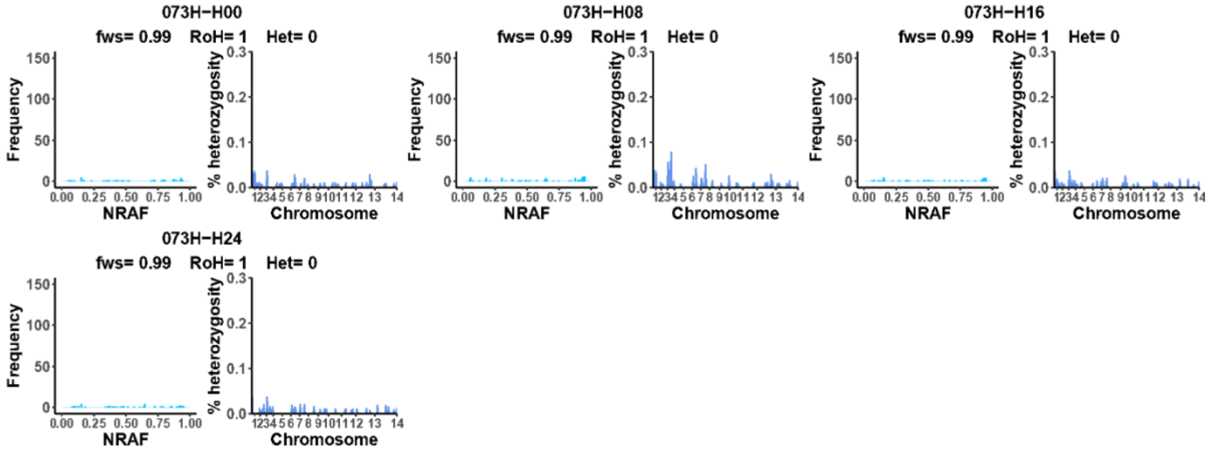
073H−H00
fws= 0.99 RoH= 1 Het= 0
073H−H08
fws= 0.99 RoH= 1 Het= 0
073H−H16
fws= 0.99 RoH= 1 Het= 0
073H−H24
fws= 0.99 RoH= 1 Het= 0
Frequency
NRAF
% heterozygosity
Chromosome

Supplementary Data 2. Individuals parasite clearance curve by timepoints post-antimalarial treatment. Quinine (A) and artesunate (B) treated groups.

A

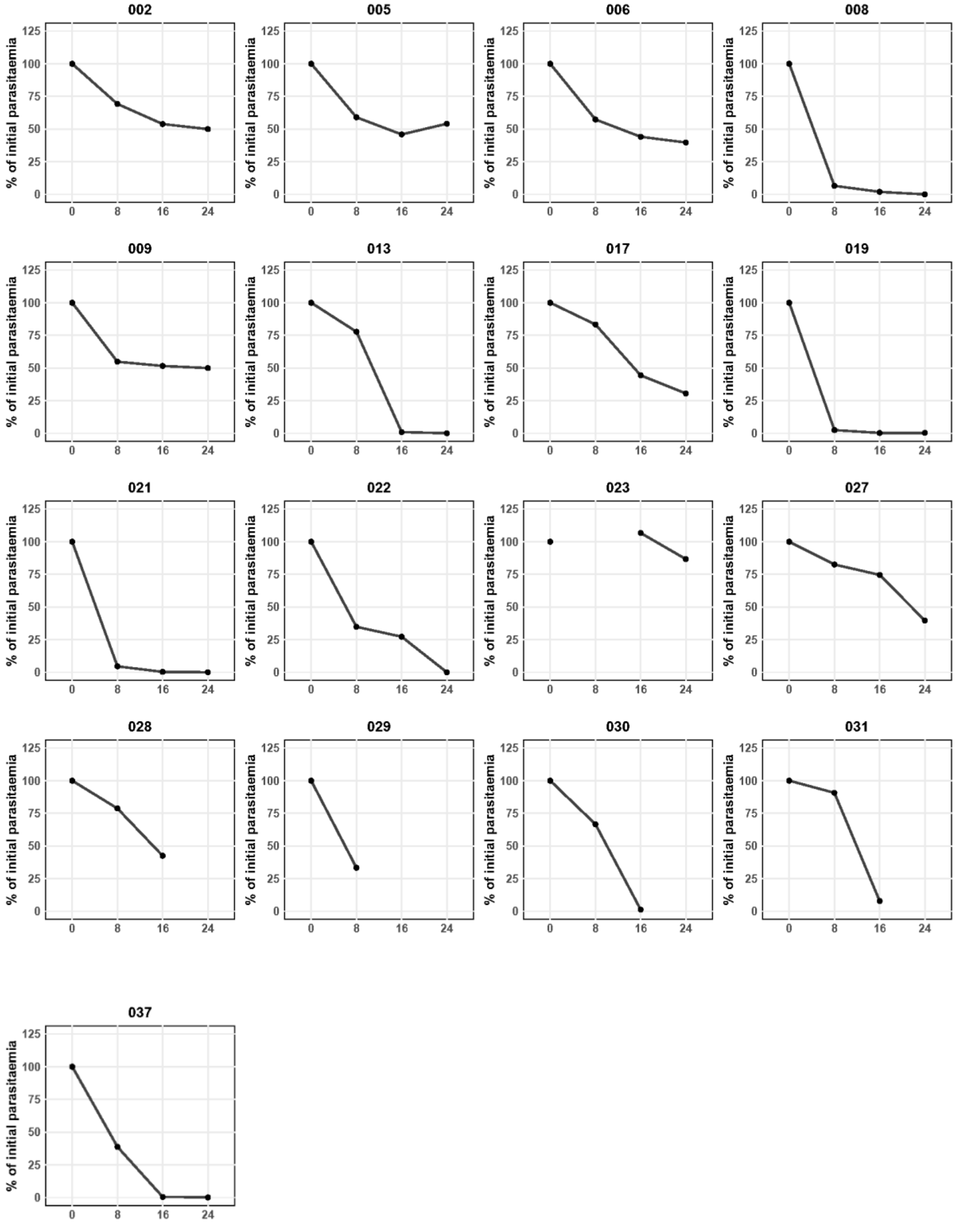

B

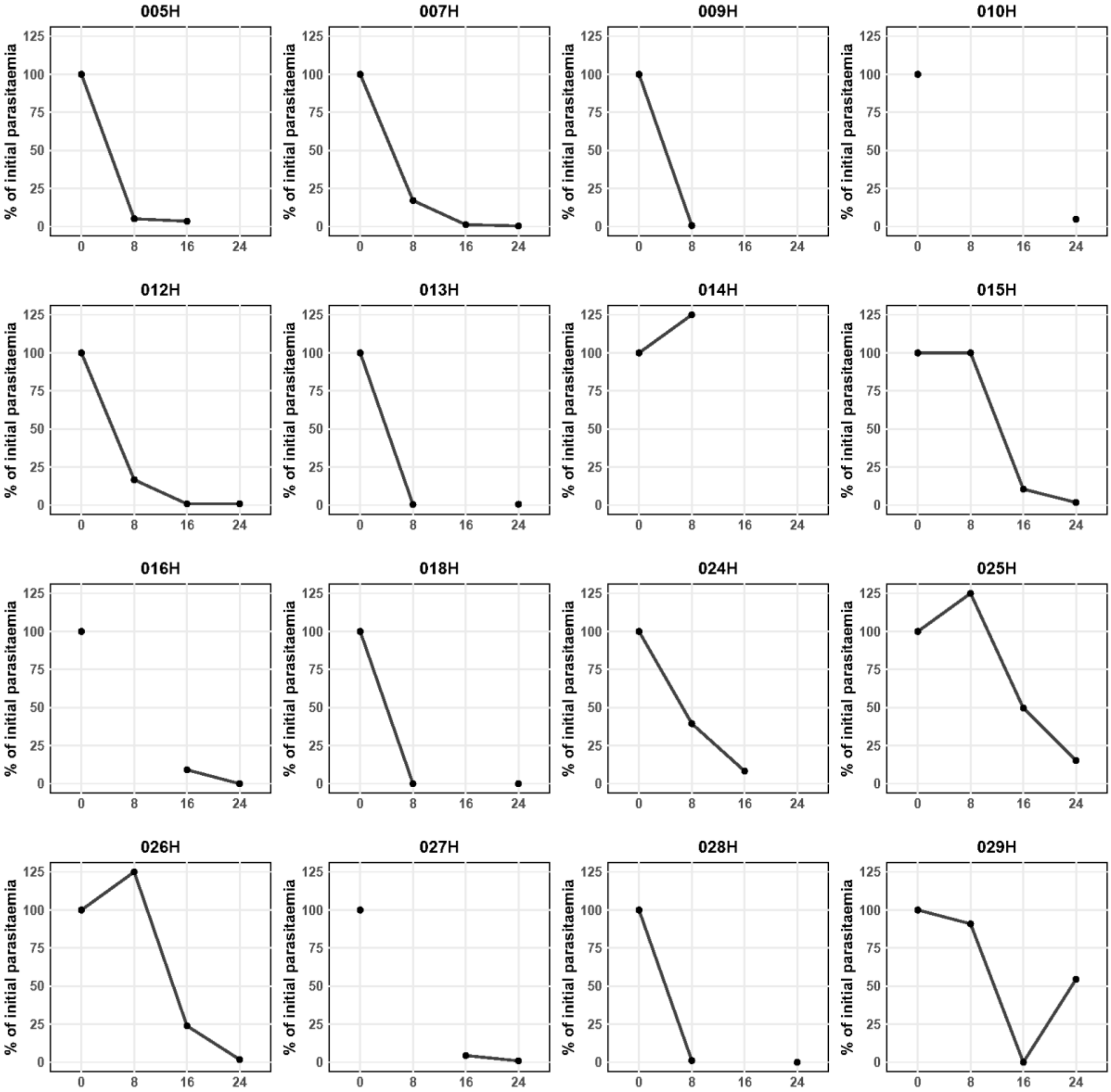
005H
007H
009H
010H
012H
013H
014H
015H
016H
018H
024H
025H
026H
027H
028H
029H
% of initial parasitaemia
0
25
50
75
100
125
0
8
16
24

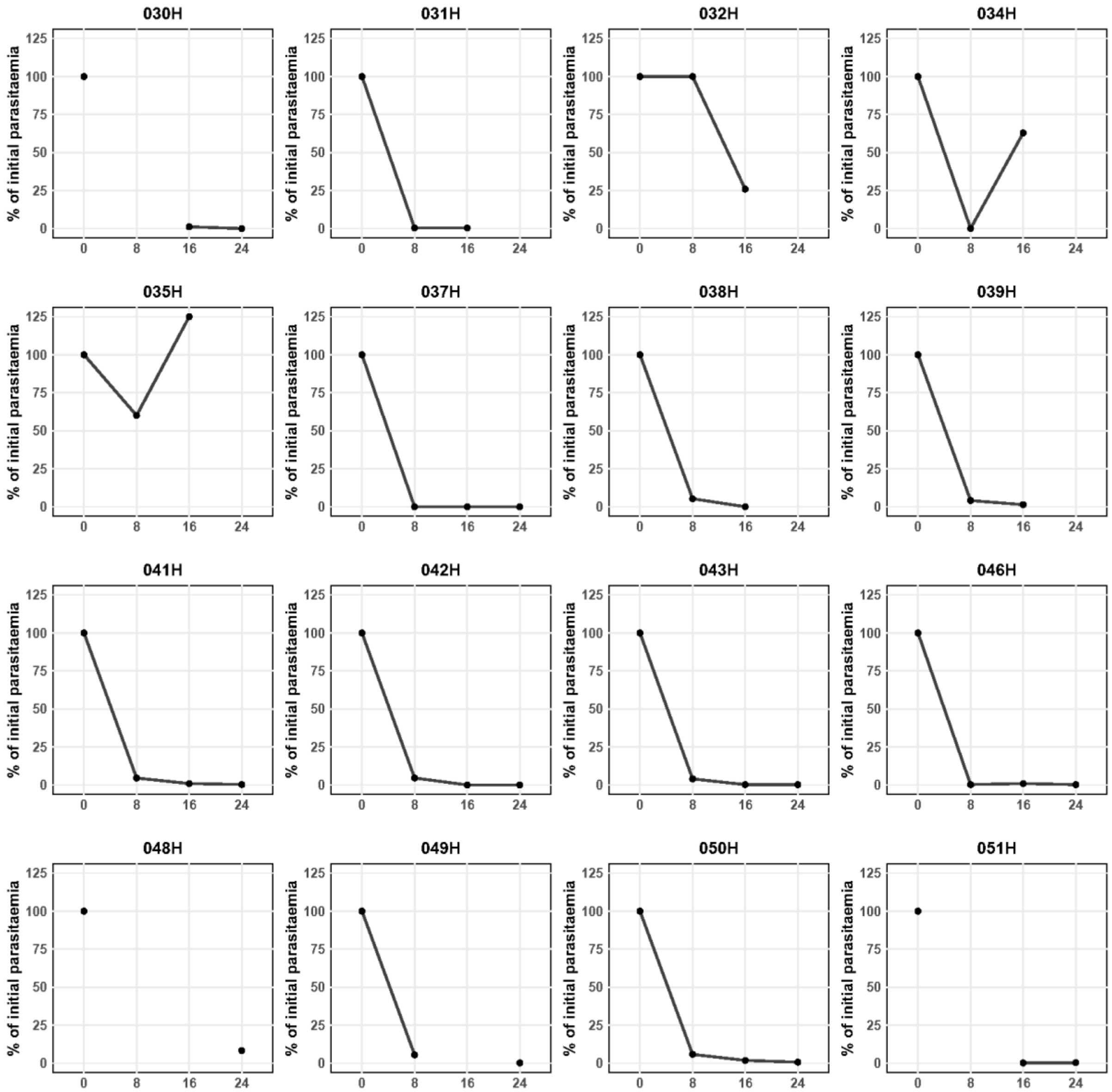
030H
031H
032H
034H
035H
037H
038H
039H
041H
042H
043H
046H
048H
049H
050H
051H
% of initial parasitaemia
0
25
50
75
100
125
0
8
16
24

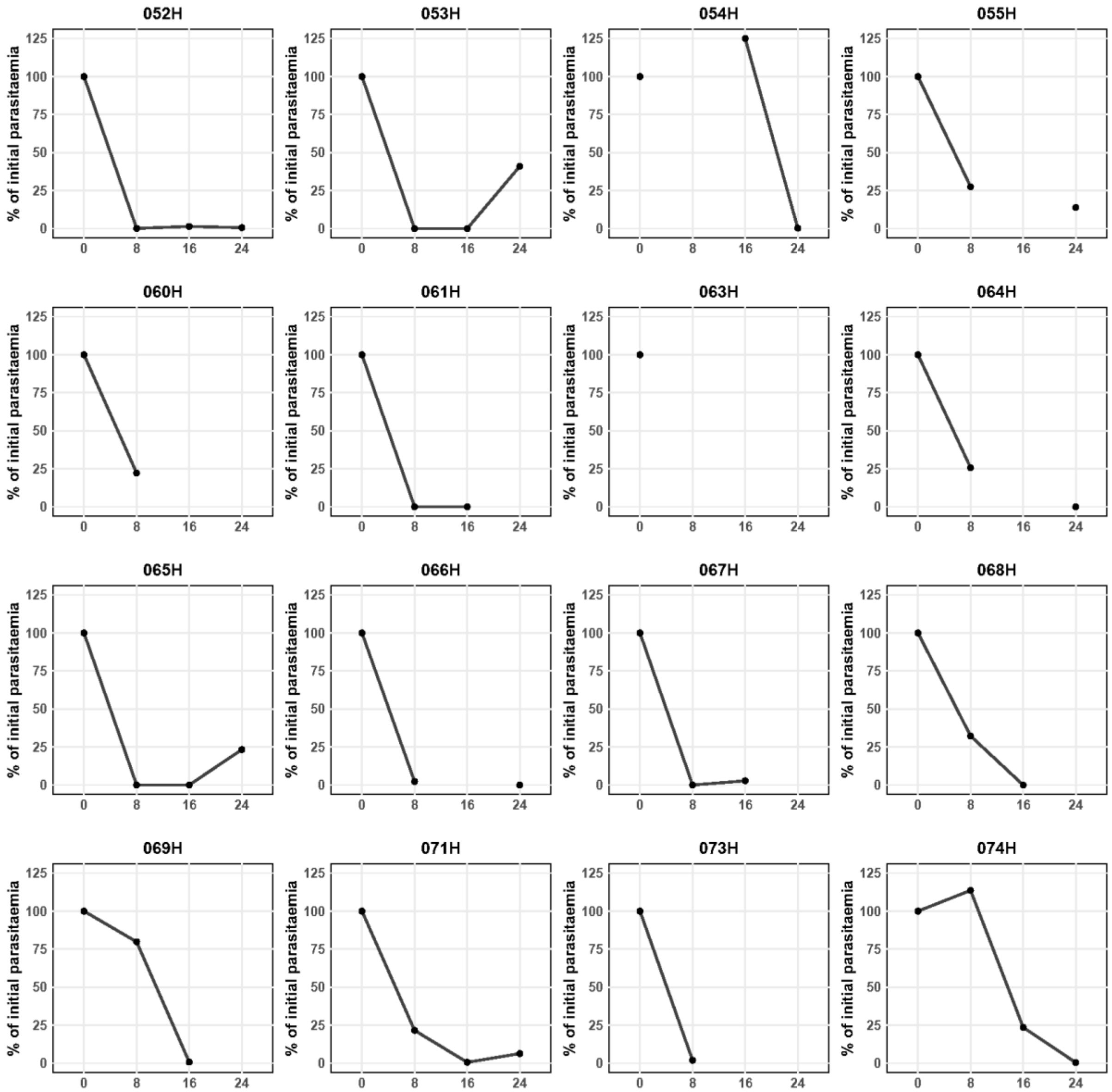

References

1. **Pearson RD, Amato R, Auburn S, Miotto O, Almagro-Garcia J, *et al.*** Genomic analysis of local variation and recent evolution in Plasmodium vivax. *Nat Genet* 2016;48:959–964.
